# LncRNA *LINK‐*A Remodels Tissue Inflammatory Microenvironments to Promote Obesity

**DOI:** 10.1002/advs.202303341

**Published:** 2023-12-25

**Authors:** Yu Chen, Hui Chen, Ying Wang, Fangzhou Liu, Xiao Fan, Chengyu Shi, Xinwan Su, Manman Tan, Yebin Yang, Bangxing Lin, Kai Lei, Lei Qu, Jiecheng Yang, Zhipeng Zhu, Zengzhuang Yuan, Shanshan Xie, Qinming Sun, Dante Neculai, Wei Liu, Qingfeng Yan, Xiang Wang, Jianzhong Shao, Jian Liu, Aifu Lin

**Affiliations:** ^1^ MOE Laboratory of Biosystem Homeostasis and Protection College of Life Sciences Zhejiang University Hangzhou Zhejiang 310058 China; ^2^ The Fourth School of Clinical Medicine Zhejiang Chinese Medical University Hangzhou Zhejiang 310053 China; ^3^ Key Laboratory of Integrated Oncology and Intelligent Medicine of Zhejiang Province Affiliated Hangzhou First People's Hospital Zhejiang University School of Medicine Hangzhou Zhejiang 310006 China; ^4^ Zhejiang University‐University of Edinburgh Institute (ZJU‐UoE Institute) University School of Medicine International Campus Zhejiang University Haining Zhejiang 314400 China; ^5^ The Children's Hospital National Clinical Research Center for Child Health Zhejiang University School of Medicine Hangzhou Zhejiang 310003 China; ^6^ Department of Cell Biology Zhejiang University School of Medicine Hangzhou Zhejiang 310058 China; ^7^ Department of Biochemistry Department of Cardiology of Second Affiliated Hospital Zhejiang University School of Medicine Hangzhou Zhejiang 313000 China; ^8^ International School of Medicine International Institutes of Medicine The 4th Affiliated Hospital of Zhejiang University School of Medicine Yiwu Zhejiang 322000 China; ^9^ Department of Cell Biology Department of General Surgery of Sir Run Run Shaw Hospital Zhejiang University School of Medicine Hangzhou Zhejiang 310016 China; ^10^ Department of Central Laboratory The First People's Hospital of Huzhou Huzhou Zhejiang 313000 China; ^11^ Cancer Center Zhejiang University Hangzhou Zhejiang 310058 China; ^12^ Hangzhou Cancer Institution Affiliated Hangzhou Cancer Hospital Zhejiang University School of Medicine Zhejiang University Hangzhou Zhejiang 310002 China; ^13^ College of Medicine and Veterinary Medicine The University of Edinburgh Edinburgh EH16 4SB UK; ^14^ Key Laboratory for Cell and Gene Engineering of Zhejiang Province Hangzhou Zhejiang 310058 China; ^15^ Future Health Laboratory Innovation Center of Yangtze River Delta Zhejiang University Jiaxing Zhejiang 314100 China; ^16^ Key Laboratory of Cancer Prevention and Intervention China National Ministry of Education Hangzhou Zhejiang 310009 China

**Keywords:** HIF1α, high‐fat diet, inflammatory microenvironment, LncRNA, obesity, therapeutic target, thermogenesis

## Abstract

High‐fat diet (HFD)‐induced obesity is a crucial risk factor for metabolic syndrome, mainly due to adipose tissue dysfunctions associated with it. However, the underlying mechanism remains unclear. This study has used genetic screening to identify an obesity‐associated human lncRNA *LINK‐A* as a critical molecule bridging the metabolic microenvironment and energy expenditure in vivo by establishing the HFD‐induced obesity knock‐in (KI) mouse model. Mechanistically, HFD *LINK‐A* KI mice induce the infiltration of inflammatory factors, including IL‐1β and CXCL16, through the *LINK‐A*/HB‐EGF/HIF1α feedback loop axis in a self‐amplified manner, thereby promoting the adipose tissue microenvironment remodeling and adaptive thermogenesis disorder, ultimately leading to obesity and insulin resistance. Notably, *LINK‐A* expression is positively correlated with inflammatory factor expression in individuals who are overweight. Of note, targeting *LINK‐A* via nucleic acid drug antisense oligonucleotides (ASO) attenuate HFD‐induced obesity and metabolic syndrome, pointing out *LINK‐A* as a valuable and effective therapeutic target for treating HFD‐induced obesity. Briefly, the results reveale the roles of lncRNAs (such as *LINK‐A*) in remodeling tissue inflammatory microenvironments to promote HFD‐induced obesity.

## Graphical abstract

This manuscript identifies a human obesity‐associated long noncoding RNA, *LINK‐A*, by generating a *de novo* knock‐in（KI） mouse model to demonstrate it as a critical molecule bridging the metabolic microenvironment and energy expenditure in vivo via establishing a high‐fat diet (HFD)‐induced obesity KI mouse model and uncovering the mechanisms regulated by the *LINK‐A*/HB‐EGF/HIF1α feedback loop.

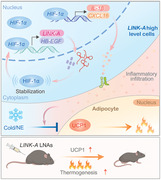

## 1 Introduction

The prevalence of obesity has rapidly increased to become a considerable public health concern worldwide,^[^ ^1^ ^]^ necessitating the development of effective therapies for obesity. Adipose tissue dysfunction associated with high sugar and fat diets is a key factor in the development of obesity and its induced metabolic diseases.^[^ ^2^, ^3^, ^4^, ^5^, ^6^ ^]^ Adipose tissue dysfunction in obesity involves functional and metabolic alterations of adipocytes (fat cells) and changes in the immune cells that reside in adipose tissue.^[^ ^7^ ^]^ Studies have revealed that excessive accumulation of triglycerides in adipocytes causes adipocyte hypertrophy. In addition, enlarged adipose tissue leads to dysregulation of adipokine secretion and increased release of free fatty acids, promoting inflammation and insulin resistance.^[^ ^7^, ^8^, ^9^, ^10^ ^]^ With further expansion of adipocytes, inadequate vascularization of adipose tissue causes hypoxia.^[^ ^11^ ^]^ Furthermore, it induces adipocyte death, triggering an innate immune response leading to increased infiltration of multiple immune cells in adipose tissue,^[^ ^12^, ^13^, ^14^, ^15^ ^]^ which secrete proinflammatory cytokines that further impair adipocyte function and internal tissue homeostasis.^[^ ^16^, ^17^, ^18^, ^19^ ^]^ Among them, interleukin 1 beta (IL‐1β) has been shown to reprogram mitochondrial metabolism in brown adipocytes by driving IL‐1 receptor (IL‐1R)‐associated kinase 2 (IRAK2)‐dependent signaling, promoting high‐fat diet (HFD)‐induced obesity and brown adipose tissue dysfunction.^[^ ^20^ ^]^ These studies highlight the contribution of adipose tissue function to obesity and associated metabolic diseases.

The inflammatory microenvironment is formed by an increase in the production and release of cytokines into the microenvironment during weight gain and remodeling of the immune cell landscape, for example, the conversion of macrophages from M2 to M1 types.^[^ ^14^ ^]^ A feed‐forward mechanism exists in obesity wherein inflamed adipose tissue stimulates the production of more monocytes, exacerbating inflammation and associated disease processes. This is because the adipose tissue‐derived S100 calcium‐binding protein A8/A9 complex (S100A8/A9) induces IL‐1β expression via toll‐like receptor 4 (TLR4)/myeloid differentiation primary response gene 88 (MyD88) pathway. IL‐1β interacts with the IL‐1R on bone marrow (BM) myeloid progenitor cells and stimulates monocyte and neutrophil production.^[^ ^21^ ^]^ In addition, a diet high in saturated and trans fats and low in fiber can directly contribute to the development of an inflammatory microenvironment.^[^ ^22^, ^23^, ^24^, ^25^, ^26^ ^]^ Saturated and trans fats can activate TLRs on immune cells and induce the release of large amounts of proinflammatory cytokines via the IκB kinase beta (IKKβ)/nuclear transcription factor‐κB (NF‐κB) or c‐Jun N‐terminal kinase 1 (JNK1)/activating protein‐1 (AP‐1) pathways.^[^ ^23^, ^27^ ^]^ Low fiber intake can also promote the development of an inflammatory microenvironment by altering the composition of the gut microbiome, leading to an increase in intestinal permeability and the release of proinflammatory factors.^[^ ^28^, ^29^ ^]^ Notably, microenvironments can also affect other organs, such as the liver, by promoting the development of nonalcoholic fatty liver disease (NAFLD), commonly associated with obesity.^[^ ^30^ ^]^ Inflammatory cytokines released from adipose tissue can promote fat accumulation in the liver, leading to liver inflammation, fibrosis, and cirrhosis.^[^ ^31^, ^32^, ^33^, ^34^, ^35^ ^]^ Overall, the inflammatory microenvironment plays a key role in the development of HFD‐induced obesity by promoting adipose tissue dysfunction, insulin resistance, and other metabolic disturbances.

Recent studies have reported that lncRNAs can regulate the inflammatory microenvironment in various tissues, including adipose tissue.^[^ ^36^, ^37^, ^38^, ^39^, ^40^ ^]^ For example, the lncRNA lnc‐DC enhances the production of proinflammatory cytokines such as IL‐6 and IL‐12 by regulating dendritic cell function.^[^ ^36^, ^38^ ^]^ In mouse blood, lncRNA‐Cox2 regulates macrophage polarization and inflammatory response by activating the cAMP‐response element binding protein (CREB)‐CCAAT/enhancer binding protein beta (C/EBPβ) signaling pathway.^[^ ^40^ ^]^ Notably, the role of lncRNAs, especially tissue‐specific lncRNAs, in HFD‐induced obesity has gained attention.^[^ ^41^, ^42^ ^]^ For example, lncRNA, lncSHGL present in mouse liver ameliorates hyperglycemia and hepatic steatosis in obese mice by recruiting heterogeneous nuclear ribonucleoprotein A1 (hnRNPA1) to activate the phosphatidylinositol 3‐kinase (PI3K)/protein kinase B (PKB) signaling pathway and inhibiting the mammalian target of rapamycin (mTOR)/sterol regulatory element binding protein‐1c (SREBP‐1c) pathway.^[^ ^41^ ^]^ However, the tissue‐specific lncRNA involved in regulating the inflammatory microenvironment and HFD‐induced obesity has not been well characterized.

Here, by constructing an obesity‐associated human lncRNA *LINK‐A* knock‐in（KI） mouse model and an HFD‐induced obesity metabolic model, we found that *LINK‐A* plays a crucial role in the metabolic microenvironment and energy consumption cross‐talk in vivo by promoting obesity and metabolic disorders induced by the HFD. Mechanistically, increased *LINK‐A* in mouse mammary epithelial cells stabilizes HIF1α by synergizing with HFD‐induced HB‐EGF, and HIF1α acts as a transcription factor that regulates the expression and release of inflammatory factors IL‐1β and CXCL16, thereby triggering inflammatory signals in the adipose tissue microenvironment. Moreover, this cytokine‐induced remodeling of the microenvironment continues through the *LINK‐A*‐activated HB‐EGF/HIF1α loop, leading to adipose tissue dysfunction and impairments in adaptive thermogenesis, ultimately leading to obesity in mice. Treating mice with antisense oligonucleotides (ASO) drugs that bind and reduce *LINK‐A* expression reduces inflammatory factors in the regional microenvironment. Furthermore, it restores adaptive thermogenesis in adipose tissue and obesity‐induced insulin resistance. Clinically, the expression of *LINK‐A* in individuals who are overweight positively correlated with the level of inflammatory factors in the regional microenvironment, indicating the clinical application of *LINK‐A* as a critical molecule involved in the cross‐talk between the regional microenvironment and metabolic diseases. In conclusion, we revealed that lncRNA might be a therapeutic target for obesity‐induced metabolic diseases. Nucleic acid drugs targeting lncRNAs may provide effective intervention strategies for obesity and obesity‐induced metabolic diseases.

## 2 Results

### 2.1 *LINK‐A* Overexpression in Mice Promotes HFD‐Induced Obesity and Insulin Resistance

**Figure 1:**
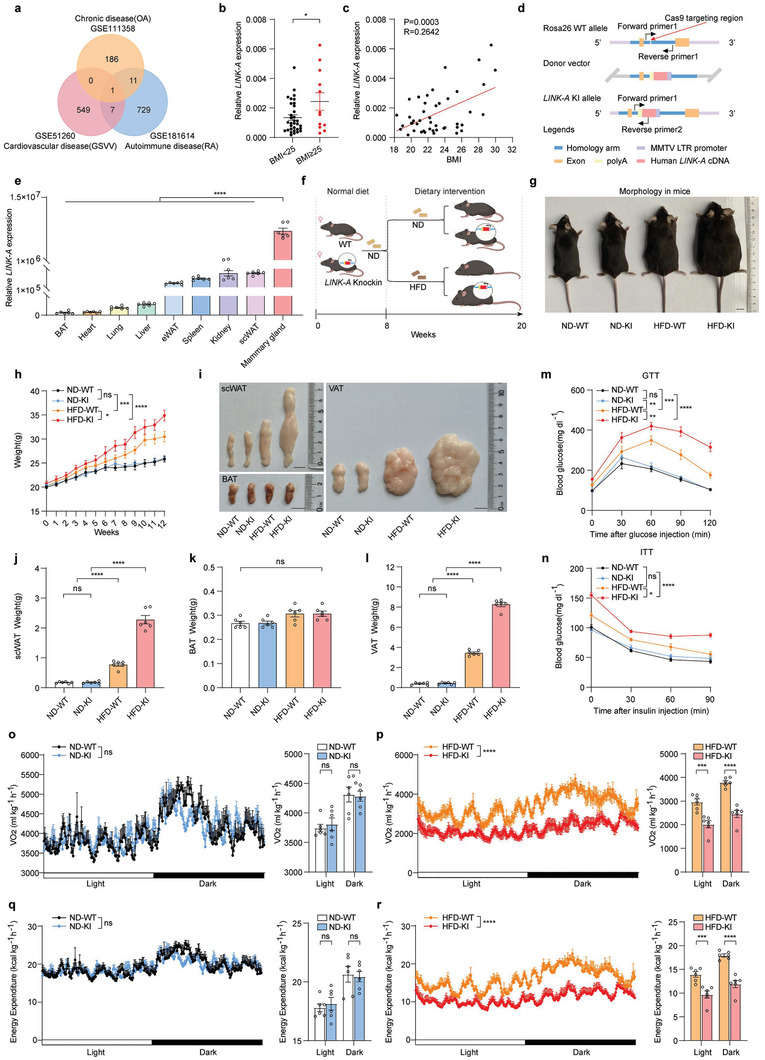
*LINK‐A* overexpression in mice promotes HFD‐induced obesity and insulin resistance a) Venn diagram of differentially expressed lncRNAs in a database of high‐risk diseases in individuals who are obesity (OA: osteoarthritis, GSE111358; GSVV: varicosis of great saphenous vein, GSE51260; RA: rheumatoid arthritis, GSE181614). b) *LINK‐A* expression was detected by qRT‐PCR in breast tissues of patients in the BMI <25 and BMI≥ 25 groups. Data presented as mean ± SEM, mammary gland (n = 33, 12), Student's t‐test, the data conform to normal or lognormal distribution, *p <0.05. c) Correlation analysis of *LINK‐A* expression in the breast tissues of patients with patient BMI. Data presented as mean ± SEM, mammary gland (n = 45), Pearson chi‐square test, *p* = 0.0003. d) Schematic diagram of *LINK‐A* KI mouse construction strategy. e) The *LINK‐A* expression levels in different tissues of *LINK‐A* KI mice were detected by qRT‐PCR. Data presented as mean ± SEM, n = 6, one‐way ANOVA, ****p <0.0001. f) Schematic diagram of the feeding strategy for mice. WT and *LINK‐A* KI mice received the dietary intervention (ND or HFD) at 8 weeks of age. g) Representative photographs of ND‐WT, ND‐KI, HFD‐WT, and HFD‐KI mice at 20 weeks of age. Scale bar: 1 cm. h) Body weight changes in ND‐WT, ND‐KI, HFD‐WT, and HFD‐KI mice after dietary intervention. Data presented as mean ± SEM, per group n = 6, two‐way ANOVA, ns = no significance, *p <0.05, ***p <0.001, ****p <0.0001. i) Representative anatomical views of adipose tissue from ND‐WT, ND‐KI, HFD‐WT, and HFD‐KI mice at 20 weeks of age. Scale bar: 1 cm. j–l) The bar graph shows the scWAT(j), BAT(k), and VAT(l) weight statistics of mice. Data presented as mean ± SEM, per group n = 6, one‐way ANOVA, ns = no significance, ****p <0.0001. m–n) At 20 weeks of age, the glucose tolerance test (GTT)(m) and insulin tolerance test (ITT)(n) were conducted on ND‐WT, ND‐KI, HFD‐WT, and HFD‐KI mice. Data presented as mean ± SEM, per group n = 6, two‐way ANOVA, ns = no significance, *p <0.05, **p <0.01, ***p <0.001, ****p <0.0001. o–p) Line graphs(left) and bar charts(right) of VO_2_ in WT and *LINK‐A* KI mice fed ND(o) or HFD(p). Data presented as mean ± SEM, per group n = 6, two‐way ANOVA, ns = no significance, ***p <0.001, ****p <0.0001. q–r) Line graphs(left) and bar charts(right) of Energy expenditure in WT and *LINK‐A* KI mice fed ND(q) or HFD(r). Data presented as mean ± SEM, per group n = 6, two‐way ANOVA, ns = no significance, ***p <0.001, ****p <0.0001.

To identify the specific lncRNAs involved in obesity, we examined lncRNA expression profiles between normal and obesity‐induced high‐risk disease tissues from multiple representative databases.^[^ ^43^, ^44^, ^45^ ^]^ Statistical analysis of RNA‐seq transcriptome profiles showed that *LINK‐A* expression was conserved among these three databases of differentially expressed lncRNAs (**Figure** **1**a). Through sequence alignment analysis of *LINK‐A* between humans and mice, we found that *LINK‐A* was a human‐specific lncRNA, poorly conserved in mice (Query cover: 4%, PhastCons score: 0.122) (Figure S1a, Supporting Information). Body mass index (BMI) is a well‐accepted way to measure fatness and thinness.^[^ ^46^, ^47^ ^]^ Therefore, we collected multiple tissue samples including adipocyte‐rich mammary gland, subcutaneous white adipose tissue (scWAT), and visceral adipose tissue (VAT) from patients and divided them into regular and overweight groups based on BMI (normal, BMI<25; overweight, BMI≥25) (Table S1, Supporting Information) to verify the relationship between *LINK‐A* and obesity. We found that the expression of *LINK‐A* was considerably increased in the overweight group compared to the normal group (*P* <0.05) (Figure 1b; Figure S1b, Supporting Information). Moreover, the *LINK‐A* expression was positively correlated with BMI (Figure 1c; Figure S1c, Supporting Information).

We have previously shown that *LINK‐A* promoted breast carcinogenesis and malignant progression.^[^ ^48^, ^49^ ^]^ To further investigate the in vivo roles of *LINK‐A*, especially in obesity, we constructed a *LINK‐A* KI mouse model to express human‐specific *LINK‐A* under the control of the mouse mammary tumor virus (MMTV)‐long terminal repeat (LTR) promoter^[^ ^50^, ^51^ ^]^ using CRISPR/Cas9 KI technology (Figure 1d). To confirm the *LINK‐A* KI, we tested the genotypes of all pregnant mice and selected homozygous *LINK‐A* KI mice for further breeding and experiments (Figure S1d, Supporting Information). We examined the *LINK‐A* expression in different mouse tissues. We found that *LINK‐A* was highly expressed in several tissues and organs, most notably in mammary tissue (Figure 1e). We then fed mice with normal diets (ND) and HFD at week 8 after birth (Figure 1f). At week 20, we observed a notable obese phenotype in *LINK‐A* KI mice compared to wild‐type (WT) mice under HFD (Figure 1g), such as the body weight (Figure 1h). It was accompanied by an increase in scWAT and VAT size and weight (Figure 1i,j,l). Simultaneously, no significant differences were observed in brown adipocyte tissue (BAT) size and weight (Figure 1i,k). Consistently, histological analysis revealed larger lipid droplets in the scWAT, BAT, and VAT of HFD‐KI mice (Figure S1e–h, Supporting Information). As expected, HFD‐KI mice had decreased glucose tolerance and increased insulin resistance relative to HFD‐WT mice (Figure 1m,n).

The etiology of obesity has been widely attributed to a dysregulation in energy metabolism.^[^ ^52^ ^]^ We further investigated the effect of *LINK‐A* overexpression on appetite in mice. Knock‐in of *LINK‐A* had no significant effect on food intake in mice compared to WT mice (Figure S1i, Supporting Information). Next, we examined the energy expenditure of mice using a metabolic cage. No significant differences were observed under ND conditions (Figure 1o,q; Figure S1j, Supporting Information). The results showed that the total oxygen consumption (VO_2_) and energy expenditure of *LINK‐A* KI mice were considerably lower than those of WT mice under HFD (Figure 1p,r). In addition, the respiratory exchange ratio (RER) was notably elevated in HFD‐KI mice relative to HFD‐WT mice, suggesting that HFD‐KI mice prefer carbohydrate fuels over lipids and have a reduced capacity for fat consumption (*P* <0.01) (Figure S1k, Supporting Information). The data presented in this study indicate the positive association between obesity and reduced energy expenditure in HFD‐KI mice. These findings imply that the overexpression of *LINK‐A* promoted obesity in vivo by suppressing energy expenditure.

### 2.2 *LINK‐A* Overexpression Reduces Adaptive Thermogenic Function in Mice Under HFD, Contributing the HFD‐Induced Obesity

**Figure 2:**
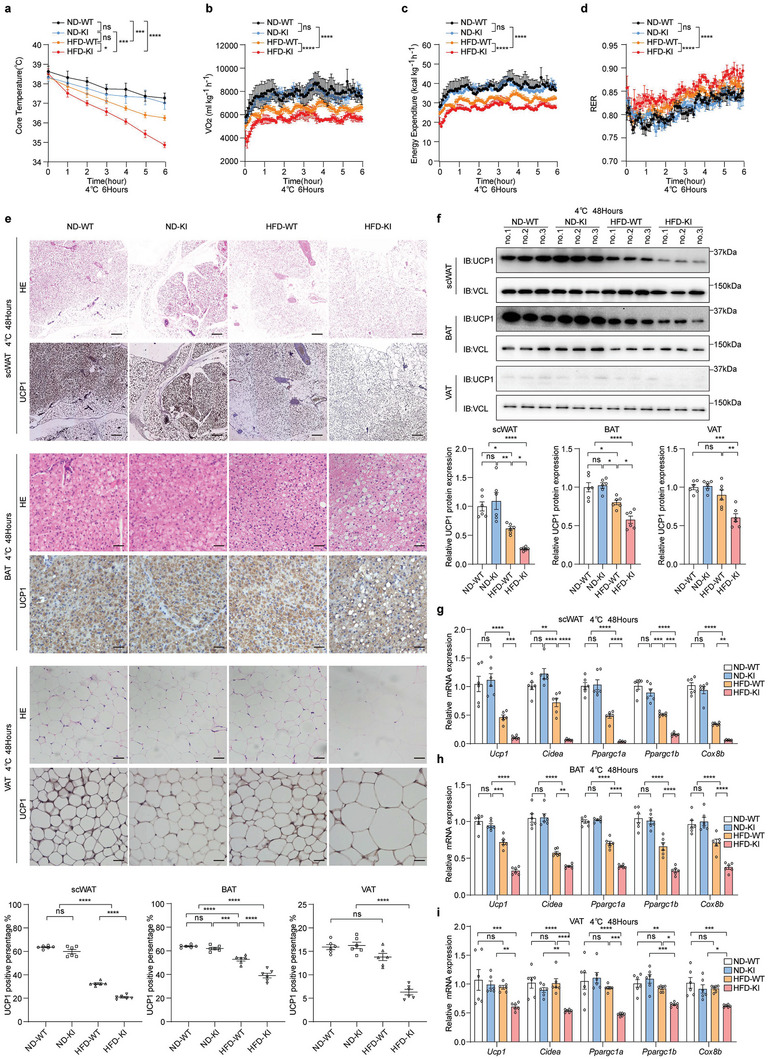
*LINK‐A* overexpression reduces adaptive thermogenic function in mice under HFD, contributing the HFD‐induced obesity a) Core body temperature of ND‐WT, ND‐KI, HFD‐WT, and HFD‐KI during a 6 h cold challenge (fasting at 30 °C for 16 h and then transfer to 4 °C). Data presented as mean ± SEM, per group n = 6, two‐way ANOVA, ns = no significance, *p <0.05, ***p <0.001, ****p <0.0001. b–d) VO_2_(b), Energy expenditure(c), and RER(d) of ND‐WT, ND‐KI, HFD‐WT, and HFD‐KI mice at cold temperature (4 °C). Data presented as mean ± SEM, per group n = 6, two‐way ANOVA, ns = no significance, ****p <0.0001. e) Representative images of hematoxylin‐eosin (H&E)‐stained sections and immunohistochemistry (IHC) of different adipose tissues. The UCP1 levels were analyzed using ImageJ. Scale bar: scWAT(200 µm), BAT(50 µm), and VAT(50 µm). Data presented as mean ± SEM, per group n = 6, one‐way ANOVA, ns = no significance, ***p <0.001, ****p <0.0001. f) Immunoblot analysis of UCP1 protein levels in ND‐WT, ND‐KI, HFD‐WT, and HFD‐KI mice housed at 4 °C for 48 h. The UCP1 protein was quantified using ImageJ. Data presented as mean ± SEM, per group n = 6, one‐way ANOVA, ns = no significance, *p <0.05, **p <0.01, ***p <0.001, ****p <0.0001. g–i) qRT‐PCR analysis of thermogenic genes in scWAT(g), BAT(h), and VAT(i) of ND‐WT, ND‐KI, HFD‐WT, and HFD‐KI mice housed at 4 °C for 48 h. Data presented as mean ± SEM, per group n = 6, two‐way ANOVA, ns = no significance, *p <0.05, **p <0.01, ***p <0.001, ****p <0.0001.

Obesity and lower energy expenditures in *LINK‐A* KI mice under HFD feeding conditions may be because of the reduced adaptive thermogenic function. Therefore, we exposed mice to a cold environment to determine the effect of *LINK‐A* on adaptive thermogenesis, one of the considerable contributors to energy expenditure.^[^ ^53^ ^]^ Low ambient temperatures stimulate heat production in BAT and beige adipocytes, acquiring the additional thermogenic capacity to maintain core body temperature.^[^ ^54^ ^]^ Mice were kept under thermoneutrality (30 °C) and fasted for 16 h before being transferred to pre‐cooled metabolic cages (4 °C) without access to food. Subsequently, their body temperatures were monitored hourly for 6 h to eliminate the effect of feeding situation and food composition on the thermogenic capacity of mice.^[^ ^55^ ^]^ HFD‐KI mice were found to have considerably reduced thermoregulatory capacity during a 6 h cold challenge (**Figure** **2**a). Metabolic cage results showed lower VO_2_ and energy expenditure in the cold environment (4 °C) in the HFD‐KI mice compared to those in other groups, suggesting that the HFD‐KI mice produced less heat and had a reduced capacity for adaptive thermogenesis (Figure 2b,c). In addition, the HFD‐KI mice had higher RER than other groups, suggesting that HFD‐KI mice had a reduced ability to consume fat (Figure 2d).

BAT and scWAT are the two primary adipose tissues responsible for non‐shivering adaptive thermogenesis under cold challenge. When exposed to cold conditions, scWAT was converted to a brown phenotype called scWAT brown.^[^ ^54^ ^]^ Brown scWAT begins to express uncoupling protein‐1 (UCP1), a specific marker of BAT, making scWAT more susceptible to heat production and increasing the body's energy expenditure. Following the cold challenge, scWAT browning was reduced in HFD‐KI mice compared to other groups (Figure 2e), indicating a reduced thermogenic capacity triggered by browning under cold conditions. Following cold exposure for 48 h, the UCP1 protein expression was reduced in scWAT, BAT, and VAT of HFD‐KI mice compared to that in other groups (Figure 2f). Moreover, the mRNA expression levels of key thermogenic genes, including *Ucp1*, cell death‐inducing DFFA‐like effector a (*Cidea*), peroxisome proliferative activated receptor gamma coactivator 1 alpha (*Ppargc1a*), peroxisome proliferative activated receptor gamma coactivator 1 beta (*Ppargc1b*), and cytochrome c oxidase subunit VIIIb (*Cox8b*), were also reduced (Figure 2g–i). In conclusion, these findings showed that low‐temperature‐induced adipose tissue thermogenesis was impaired in *LINK‐A* KI mice under HFD conditions.

### 2.3 *LINK‐A* Overexpression Reduces Adaptive Thermogenesis in HFD‐Fed Mice by Remodeling the Regional Inflammatory Microenvironment

**Figure 3:**
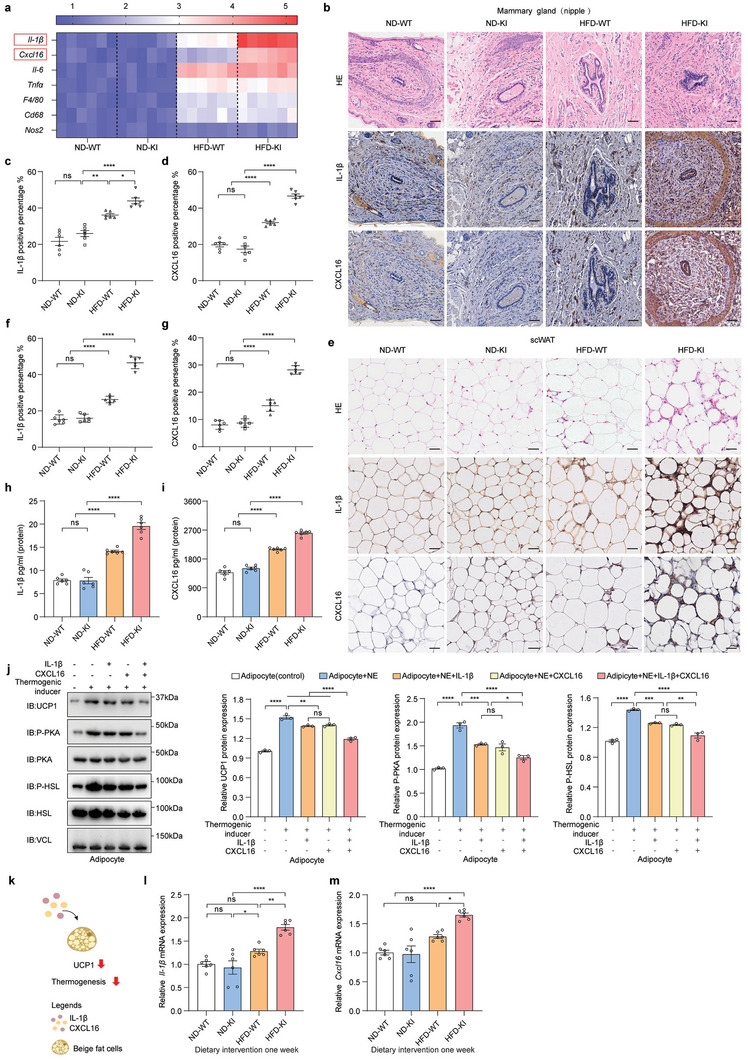
*LINK‐A* overexpression reduces adaptive thermogenesis in HFD‐fed mice by remodeling the regional inflammatory microenvironment a) Heatmap of inflammatory factor mRNA expression levels in the mammary glands of ND‐WT, ND‐KI, HFD‐WT, and HFD‐KI mice detected by qRT‐PCR. per group n = 6. b–d) Representative images of H&E‐stained sections and IHC of mammary glands(b). The IL‐1β(c) or CXCL16(d) protein levels were analyzed using ImageJ. Scale bar: 50 µm. Data presented as mean ± SEM, per group n = 6, one‐way ANOVA, ns = no significance, *p <0.05, **p <0.01, ****p <0.0001. e–g) Representative images of H&E‐stained sections and IHC of scWAT(e). The IL‐1β(f) or CXCL16(g) protein levels were analyzed using ImageJ. Scale bar: 50 µm. Data presented as mean ± SEM, per group n = 6, one‐way ANOVA, ns = no significance, ****p <0.0001. h–i) Mouse plasma concentrations of IL‐1β(h) and CXCL16(i) were measured by ELISA. Data presented as mean ± SEM, per group n = 6, one‐way ANOVA, ns = no significance, ****p <0.0001. j) The protein levels of UCP1, P‐PKA, PKA, P‐HSL, and HSL in BMSCs‐derived beige adipocytes treatment using IL‐β or/and CXCL16 cytokines by immunoblot analysis, and the protein levels were quantified using ImageJ. Data presented as mean ± SEM, pooled data from three independent experiments, one‐way ANOVA, ns = no significance, *p <0.05, **p <0.01, ***p <0.001, ****p <0.0001. k) Schematic diagram of the effect of inflammatory factors IL‐1β and CXCL16 on adipose thermogenesis. l–m) Following one week of dietary intervention, *IL‐1β*(l) and *Cxcl16*(m) mRNA expression levels of the mammary gland in WT or *LINK‐A* KI mice were detected by qRT‐PCR. Data presented as mean ± SEM, per group n = 6, one‐way ANOVA, ns = no significance, *p <0.05, **p <0.01, ****p <0.0001.

Altered adipose tissue functions in most patients with obesity are associated with chronic inflammation‐induced immune cell infiltration and local microenvironmental remodeling.^[^ ^56^ ^]^ A large amount of adipose tissue surrounds the mammary gland in mice.^[^ ^57^ ^]^ Therefore, we hypothesized that impaired tissue thermogenesis in *LINK‐A* KI mice under HFD conditions was caused by the dysregulated inflammatory microenvironment. We examined the expression of inflammatory factors in mammary tissue with the highest *LINK‐A* levels and found that proinflammatory factors were considerably higher in the mammary tissue of the HFD group than in the ND group (**Figure** **3**a; Figure S2a–g, Supporting Information). Notably, *IL‐1β* and *CXCL16* were considerably higher in the mammary glands of HFD‐KI mice than in HFD‐WT mice (Figure 3a; Figure S2a–g, Supporting Information). Although similar trends were observed in other adipose tissues (Figure S2h,i, Supporting Information), we focused on the mammary tissues with the highest expression of *LINK‐A*. The immunohistochemistry (IHC) results showed increased IL‐1β and CXCL16 infiltration in the HFD‐KI mammary gland tissue (near the nipple) and altered microenvironment (Figure 3b–d). No significant changes were observed in the expression of IL‐6 and tumor necrosis factor‐alpha (TNFα) (Figure S2j–l, Supporting Information). The scWAT surrounding the mammary glands of HFD‐KI mice also exhibited inflammatory factor infiltration (Figure 3e–g). As *LINK‐A* was expressed in various tissues and organs (Figure 1e), we examined IL‐1β and CXCL16 in mouse plasma and found that they were increased in HFD‐KI mice (Figure 3h,i). These results suggest that *LINK‐A* may trigger the expression of inflammatory factors in the microenvironment.

Not much has been reported about the role of IL‐1β and CXCL16 in regulating the thermogenic effects in obesity. Therefore, we treated beige adipocytes with IL‐1β and CXCL16 cytokines. Although the thermogenic effects of Norepinephrine (NE) on beige adipocytes were not affected by either IL‐1β or CXCL16 (Figure 3j), the simultaneous treatment of IL‐1β and CXCL16 reduced the expression levels of UCP1, phosphorylated protein kinase A (P‐PKA), and its downstream lipoprotein phosphorylated hormone‐sensitive lipase (P‐HSL) as well as the thermogenesis‐related genes (Figure 3j; Figure S2m, Supporting Information). NE is the inducer promoting non‐shivering thermogenesis by activating PKA, and thus the downstream lipolytic protein HSL through the regulation of multiple intracellular activities.^[^ ^58^ ^]^ These findings suggest that *LINK‐A* in HFD‐KI mice may trigger infiltration of local inflammatory factors IL‐1β or CXCL16 to reduce adaptive thermogenesis in mice (Figure 3k).

To clarify the sequence of inflammatory factor expression and obesity, we examined the expression of *IL‐1β* and *CXCL16* in mammary tissue of mice after 1 week of HFD before the notable difference in body weight became evident. We found that the levels of *IL‐1β* and *CXCL16* were upregulated in the mammary glands of mice at the pre‐obesity stage (Figure 3l,m). To summarize, the activated inflammatory microenvironment, such as increased expression of IL‐1β and CXCL16, may promote obesity in *LINK‐A* overexpressing mice under HFD by affecting adipocyte thermogenesis.

### 2.4 *LINK‐A* Overexpression Induces Inflammatory Factor Expression by Stabilizing HIF1α Through the HFD‐Induced HB‐EGF

**Figure 4:**
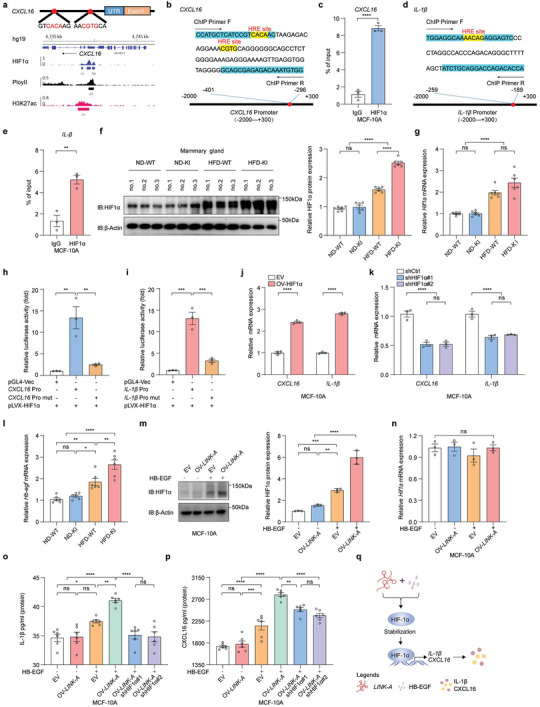
*LINK‐A* overexpression induces inflammatory factor expression by stabilizing HIF1α through the HFD‐induced HB‐EGF a) Schematic diagram of HRE site at *CXCL16* promoters(up) and HIF1α binds the *CXCL16* promotor region by HIF1α ChIP‐seq database analysis(down). b) *CXCL16* ChIP‐qPCR primer sequences(blue) and HRE site sequences (yellow). c) The interaction between HIF1α and *CXCL16* promoter in MCF‐10A was verified by ChIP‐qPCR assay. Data presented as mean ± SEM, pooled data from three independent experiments, unpaired t‐test, ****p <0.0001. d) *IL‐1β* ChIP‐qPCR primer sequences(blue) and HRE site sequences(yellow). e) The interaction between HIF1α and *IL‐1β* promoter in MCF‐10A was verified by ChIP‐qPCR assay. Data presented as mean ± SEM, pooled data from three independent experiments, unpaired t‐test, **p <0.01. f) Immunoblot analysis of HIF1α protein levels in the mammary gland of ND‐WT, ND‐KI, HFD‐WT, and HFD‐KI mice. The HIF1α protein was quantified using ImageJ. Data presented as mean ± SEM, per group n = 6, one‐way ANOVA, ns = no significance, ****p <0.0001. g) qRT‐PCR analysis of *Hif1α* mRNA in mammary glands of ND‐WT, ND‐KI, HFD‐WT, and HFD‐KI mice. Data presented as mean ± SEM, per group n = 6, one‐way ANOVA, ns = no significance, ****p <0.0001. h) The interaction between HIF1α and *CXCL16* promoter (Pro) in MCF‐10A was verified by a Dual‐Luciferase reporter assay. The *CXCL16* Pro mutant (mut) site contains HRE(b). Data presented as mean ± SEM, pooled data from three independent experiments, one‐way ANOVA, **p <0.01. i) The interaction between HIF1α and *IL‐1β* promoter in MCF‐10A was verified by a Dual‐Luciferase reporter assay. The *IL‐1β* Pro mut site contains HRE(d). Data presented as mean ± SEM, pooled data from three independent experiments, one‐way ANOVA, ***p <0.001. j) The mRNA levels of *CXCL16*, *IL‐1β* in MCF‐10A empty vector (EV) or HIF1α overexpression (OV‐HIF1α) cells were detected by qRT‐PCR. Data presented as mean ± SEM, pooled data from three independent experiments, two‐way ANOVA, ****p <0.0001. k) The mRNA levels of *CXCL16*, *IL‐1β* in MCF‐10A shControl (shCtrl) or shHIF1α (shHIF1α#1, shHIF1α#2) cells by qRT‐PCR. Data presented as mean ± SEM, pooled data from three independent experiments, two‐way ANOVA, ns = no significance, ****p <0.0001. l) qRT‐PCR analysis of *Hb‐egf* mRNA in mammary glands of ND‐WT, ND‐KI, HFD‐WT, and HFD‐KI mice. Data presented as mean ± SEM, per group n = 6, one‐way ANOVA, ns = no significance, *p <0.05, **p <0.01, ****p <0.0001. m) The protein level of HIF1α in MCF‐10A EV or *LINK‐A* overexpression (OV‐*LINK‐A*) cells with or without HB‐EGF stimulation was detected by immunoblot analysis, and the HIF1α protein was quantified using ImageJ. Data presented as mean ± SEM, pooled data from three independent experiments, one‐way ANOVA, ns = no significance, **p <0.01, ***p <0.001, ****p <0.0001. n) The mRNA level of *HIF1α* in MCF‐10A EV or *LINK‐A* overexpression cells with or without HB‐EGF stimulation was detected by qRT‐PCR. Data presented as mean ± SEM, pooled data from three independent experiments, one‐way ANOVA, ns = no significance. o,p) The concentrations of IL‐1β(o), and CXCL16(p) in the culture medium were measured by ELISA. Data presented as mean ± SEM, pooled data from six independent experiments, one‐way ANOVA, ns = no significance, *p <0.05, **p <0.01, ***p <0.001, ****p <0.0001. q) Schematic diagram of *LINK‐A* promotes the expression of IL‐1β and CXCL16 by stabilizing their transcription factors HIF1α.

To identify the regulators of IL‐1β and CXCL16, we analyzed the transcription factor bindings on their promoter regions using the published ChIP‐seq databases. First, we found that the normoxic hypoxia‐inducible factor‐1α (HIF1α) ChIP‐seq database (GSM1462475) showed its binding sites in *CXCL16* promoter regions (**Figure** **4**a). Furthermore, the gene encoding IL‐1β contains a conserved hypoxia response element (HRE) site in its promoter, leading to increased expression in response to HIF1α stabilization.^[^ ^59^ ^]^ ChIP‐qPCR also verified that HIF1α binds to the promoters of CXCL16 and IL‐1β in MCF‐10A cells (Figure 4b–e). Next, we examined the levels of HIF1α in the mammary tissues of mice. The results showed that HIF1α protein levels were considerably increased in the mammary glands of HFD‐KI mice compared to HFD‐WT (Figure 4f). Moreover, HIF1α mRNA levels were not affected by the *LINK‐A* overexpression (Figure 4g). These suggested that the HIF1α protein, stabilized by *LINK‐A* overexpression, can regulate IL‐1β and CXCL16.

To investigate the regulation of HIF1α on the expression of IL‐1β and CXCL16, luciferase reporter gene assays were performed in HEK‐293T cells. It showed that HIF1α overexpression considerably increased the transcriptional activity of the IL‐1β and CXCL16 promoters. In contrast, mutant promoters containing the deleted HRE binding motifs exhibited a lower transcriptional activity (Figure 4h,i). Further, the overexpression of HIF1α in MCF‐10A cells upregulated *IL‐1β* and *CXCL16* mRNA levels (Figure 4j; Figure S3a, Supporting Information); *IL‐1β* and *CXCL16* mRNA levels were reduced in HIF1α knockdown cells (Figure 4k; Figure S3b, Supporting Information). In conclusion, our experiments demonstrated that *LINK‐A* overexpression induced inflammatory factor (e.g., IL‐1β and CXCL16) expression in HFD‐fed mice by stabilizing HIF1α.

Next, we aimed to understand how HIF1α protein was stabilized. Our previous study had shown that *LINK‐A* and heparin‐binding epidermal growth factor (HB‐EGF) together promoted the stabilization of HIF1α protein in breast cancer cells.^[^ ^48^ ^]^ Therefore, we examined HB‐EGF expression in the mammary tissue of mice. We found that HFD considerably increased the level of *Hb‐egf* in the mammary glands of mice (Figure 4l). Furthermore, MCF‐10A cells overexpressing *LINK‐A* upon HB‐EGF stimulation enhanced HIF1α protein stability without altering its mRNA level (Figure 4m,n). Moreover, the synergistic effect of HB‐EGF and *LINK‐A* in inducing the secretion of IL‐1β and CXCL16 in the culture medium was dependent on HIF1α (Figure 4o,p).

The stabilized HIF1α indicated that the HIF1α pathway was activated in HFD‐KI mice. Therefore, we examined the expression of the other downstream genes of HIF1α in the mammary glands of mice. We found that they were upregulated in the mammary glands of HFD‐KI mice (Figure S3c–j, Supporting Information). Furthermore, it showed that the activation of the HIF1α pathway was more evident in the mammary glands of HFD‐KI mice than in HFD‐WT. The above results suggest that *LINK‐A* stabilized HIF1α protein under HFD by increasing HB‐EGF to fully activate the HIF1α pathway (Figure 4q).

### 2.5 HIF1α Positively Regulated *LINK‐A* and HB‐EGF to Form an Activated Loop Pathway of *LINK‐A*/HB‐EGF/HIF1α

**Figure 5:**
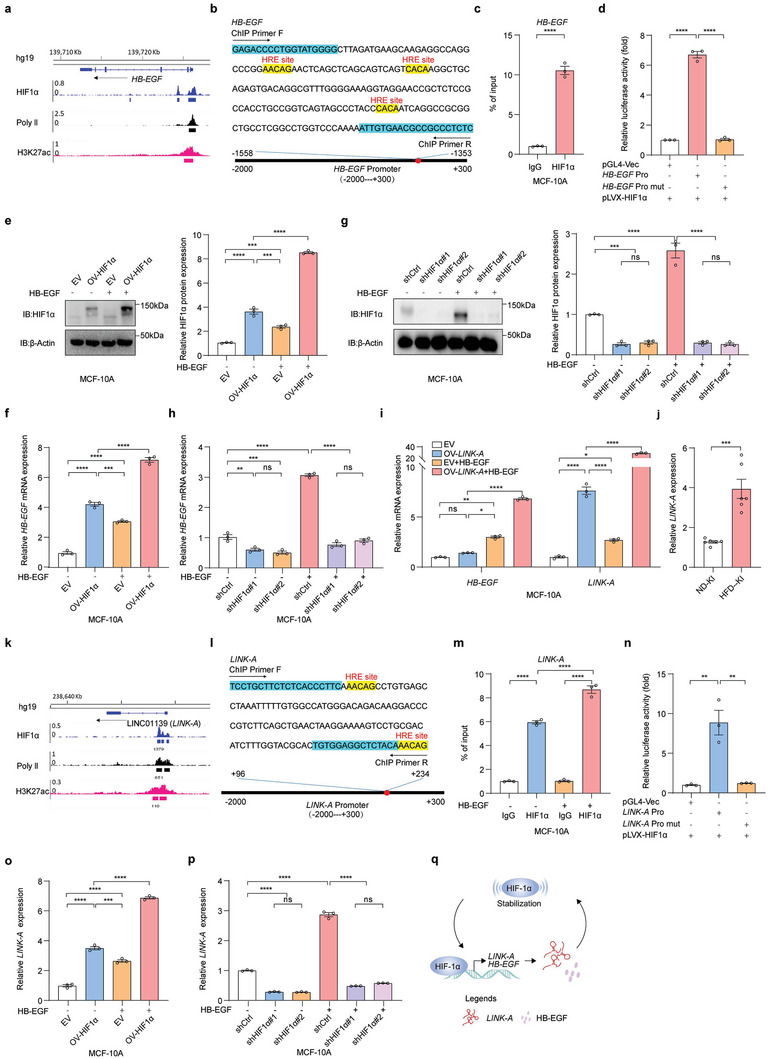
HIF1α positively regulated *LINK‐A* and HB‐EGF to form an activated loop pathway of *LINK‐A*/HB‐EGF/HIF1α a) HIF1α binds the *HB‐EGF* promotor region by HIF1α ChIP‐seq database analysis. b) *HB‐EGF* ChIP‐qPCR primer sequences(blue) and HRE site sequences(yellow). c) The interaction between HIF1α and *HB‐EGF* in MCF‐10A was verified by ChIP‐qPCR assay. Data presented as mean ± SEM, pooled data from three independent experiments, unpaired t‐test, ****p <0.0001. d) The interaction between HIF1α and *HB‐EGF* promoter in MCF‐10A was verified by a Dual‐Luciferase reporter assay. The *HB‐EGF* Pro mut site contains HRE(b). Data presented as mean ± SEM, pooled data from three independent experiments, one‐way ANOVA, ****p <0.0001. e) The protein level of HIF1α in MCF‐10A EV or HIF1α overexpression cells with or without HB‐EGF stimulation was detected by immunoblot analysis, and the HIF1α protein was quantified using ImageJ. Data presented as mean ± SEM, pooled data from three independent experiments, one‐way ANOVA, ***p <0.001, ****p<0.0001. f) The mRNA level of *HB‐EGF* in MCF‐10A EV or HIF1α overexpression cells with or without HB‐EGF stimulation was detected by qRT‐PCR. Data presented as mean ± SEM, pooled data from three independent experiments, one‐way ANOVA, ***p <0.001, ****p <0.0001. g) The protein level of HIF1α in MCF‐10A shCtrl or shHIF1α cells with or without HB‐EGF stimulation was detected by immunoblot analysis, and the HIF1α protein was quantified using ImageJ. Data presented as mean ± SEM, pooled data from three independent experiments, one‐way ANOVA, ns = no significance, ***p <0.001, ****p <0.0001. h) The mRNA level of *HB‐EGF* in MCF‐10A shCtrl or shHIF1α cells with or without HB‐EGF stimulation by qRT‐PCR. Data presented as mean ± SEM, pooled data from three independent experiments, one‐way ANOVA, ns = no significance, **p <0.01, ***p <0.001, ****p <0.0001. i) The mRNA levels of *HB‐EGF* and *LINK‐A* in MCF‐10A EV or *LINK‐A* overexpression cells with or without HB‐EGF stimulation were detected by qRT‐PCR. Data presented as mean ± SEM, pooled data from three independent experiments, two‐way ANOVA, ns = no significance, *p <0.05, **p <0.01, ****p <0.0001. j) The *LINK‐A* expression levels of the mammary gland in ND‐KI and HFD‐KI were detected by qRT‐PCR. Data presented as mean ± SEM, per group n = 6, unpaired t‐test, ***p <0.001. k) HIF1α binds the *LINK‐A* promotor region by HIF1α ChIP‐seq database analysis. l) *LINK‐A* ChIP‐qPCR primer sequences(blue) and HRE site sequences(yellow). m) The interaction between HIF1α and *LINK‐A* in MCF‐10A was verified by ChIP‐qPCR assay. Data presented as mean ± SEM, pooled data from three independent experiments, one‐way ANOVA, ****p <0.0001. n) The interaction between HIF1α and *LINK‐A* promoter in MCF‐10A was verified by a Dual‐Luciferase reporter assay. The *LINK‐A* Pro mut site contains HRE(l). Data presented as mean ± SEM, pooled data from three independent experiments, one‐way ANOVA, **p <0.01. o) The *LINK‐A* expression levels in MCF‐10A EV or HIF1α overexpression cells with or without HB‐EGF stimulation were detected by qRT‐PCR. Data presented as mean ± SEM, pooled data from three independent experiments, one‐way ANOVA, ***p <0.001, ****p <0.0001. p) The *LINK‐A* expression levels in MCF‐10A shCtrl or shHIF1α cells with or without HB‐EGF stimulation by qRT‐PCR. Data presented as mean ± SEM, pooled data from three independent experiments, one‐way ANOVA, ns = no significance, ****p <0.0001. q) Diagram of *LINK‐A*/HB‐EGF/HIF1α loop.

We aimed to determine how *Hb‐egf* expression was induced in *LINK‐A* overexpressed mice under HFD compared with the WT (Figure 4l). By analyzing the HIF1α ChIP‐seq database, we hypothesized that HIF1α induced the *HB‐EGF* mRNA expression since HIF1α bound the *HB‐EGF* promoter regions (GSM1462475) (**Figure** **5**a). ChIP‐qPCR further demonstrated that HIF1α bound on the *HB‐EGF* promoter region (Figure 5b,c). HIF1α binding on the *HB‐EGF* promoter was considerably impaired by HRE mutation (Figure 5d). Further studies revealed that overexpression of HIF1α in MCF‐10A cells upregulated the levels of *HB‐EGF*, and HB‐EGF stimulation resulted in higher *HB‐EGF* gene expression (Figure 5e,f) and that knockdown of HIF1α decreased the transcript levels of *HB‐EGF* (Figure 5g,h). Moreover, HB‐EGF stimulation leading to higher *HB‐EGF* gene expression was also suppressed by the HIF1α knockdown (Figure 5g,h). Therefore, HB‐EGF was positively regulated by HIF1α by binding on its promoter.

We found that HB‐EGF stimulation induced *LINK‐A* expression in MCF‐10A cells (Figure 5i). This is consistent with higher HB‐EGF and *LINK‐A* levels in HFD‐KI mice than in ND‐KI mice (Figures 4l and 5j). The HIF1α ChIP‐seq database (GSM1462475) also showed the binding of HIF1α on the *LINK‐A* promoter (Figure 5k). Therefore, we hypothesized that HIF1α positively regulated the *LINK‐A* expression. ChIP‐qPCR verified that HIF1α bound to the *LINK‐A* promoter region and that HB‐EGF stimulation promoted the binding of HIF1α to *LINK‐A*, possibly due to enhanced HIF1α protein stability (Figure 5l,m). Similarly, when the HRE site was mutated, the HIF1α binding to the *LINK‐A* promoter was decreased (Figure 5n). Further studies revealed that overexpression of HIF1α in MCF‐10A cells upregulated the levels of *LINK‐A*, HB‐EGF stimulation resulted in higher *LINK‐A* gene expression (Figure 5o), and HIF1α knockdown decreased the levels of *LINK‐A*. Moreover, HB‐EGF stimulation leading to higher *LINK‐A* expression also depended on HIF1α (Figure 5p). Therefore, HIF1α positively regulates *LINK‐A* by directly binding on its promoter in the presence of HB‐EGF. In conclusion, *LINK‐A* formed a positive feedback loop with HB‐EGF/HIF1α to amplify the HIF1α signaling cascade, including its direct targets, such as *LINK‐A* and HB‐EGF (Figure 5q).

### 2.6 Activation of the *LINK‐A*/HB‐EGF/HIF1α Pathway in Mammary Cells Reduces Adipocyte Thermogenesis

**Figure 6:**
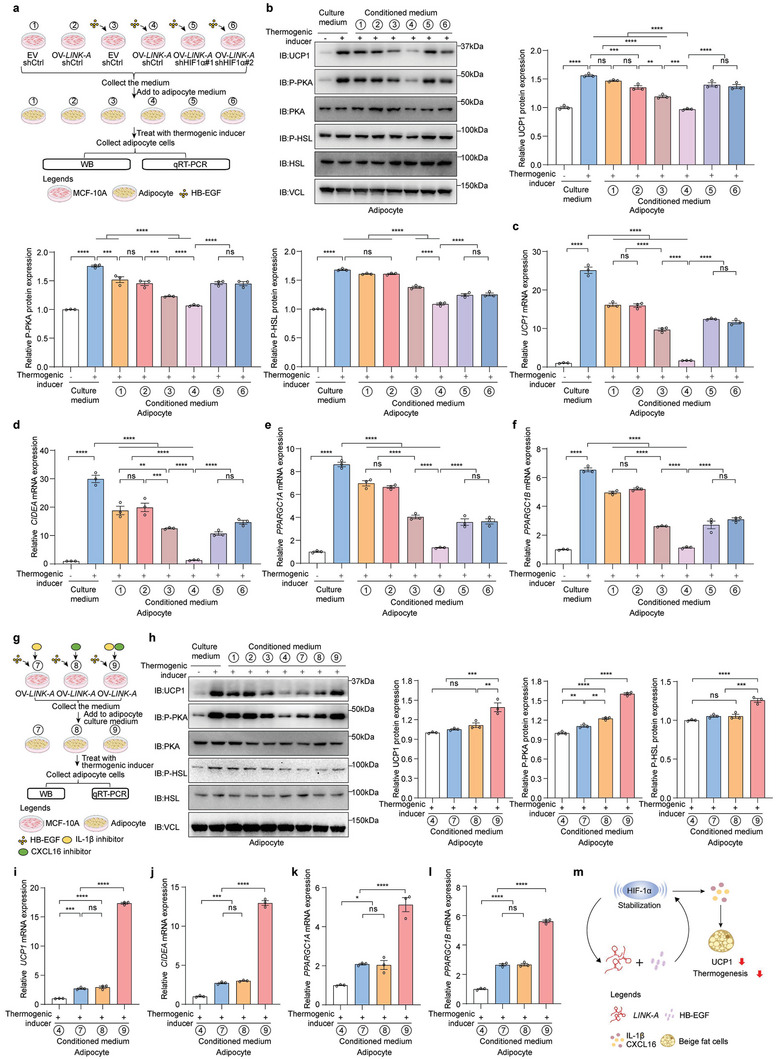
Activation of the *LINK‐A*/HB‐EGF/HIF1α pathway in mammary cells reduces adipocyte thermogenesis a) Schematic representation of bone marrow mesenchymal stem cells (BMSCs)‐derived beige adipocytes treated with MCF‐10A cells conditioned media: *LINK‐A* overexpressing MCF‐10A and control cells were stimulated with HB‐EGF, and the conditioned media was obtained to treat beige adipocytes. b) The protein levels of UCP1, P‐PKA, PKA, P‐HSL, and HSL in adipocytes treated with different conditioned media by immunoblot analysis, and the protein levels were quantified using ImageJ. Data presented as mean ± SEM, pooled data from three independent experiments, one‐way ANOVA, ns = no significance, **p <0.01, ***p <0.001, ****p <0.0001. c–f) The mRNA levels of thermogenic genes in adipocytes treated with different conditioned media by qRT‐PCR. Data presented as mean ± SEM, pooled data from three independent experiments, one‐way ANOVA, ns = no significance, **p <0.01, ***p <0.001, ****p <0.0001. g) Schematic representation of BMSCs‐derived beige adipocytes treated with MCF‐10A cells conditioned media: IL‐1β inhibitor or/and CXCL16 inhibitors add to the conditioned media of HB‐EGF‐stimulated *LINK‐A* overexpression MCF‐10A cells, respectively, and the conditioned media was obtained to treat beige adipocytes. h) The protein levels of UCP1, P‐PKA, PKA, P‐HSL, and HSL in adipocytes treated with different conditioned media by immunoblot analysis, and the protein levels were quantified using ImageJ. Data presented as mean ± SEM, pooled data from three independent experiments, one‐way ANOVA, ns = no significance, **p <0.01, ***p <0.001, ****p <0.0001. i–l) The mRNA levels of thermogenic genes in adipocytes treated with different conditioned media by qRT‐PCR. Data presented as mean ± SEM, pooled data from three independent experiments, one‐way ANOVA, ns = no significance, *p <0.05, ***p <0.001, ****p <0.0001. m) Schematic diagram of *LINK‐A* affecting adipocyte thermogenesis.

Given the inhibitory effect of downstream cytokines of HIF1α (such as CXCL16 and IL‐1β) on adipocyte thermogenesis (Figure 3j; Figure S2m, Supporting Information), we investigated the effect of activating the *LINK‐A*/HB‐EGF/HIF1α pathway in MCF‐10A cells on adipocyte thermogenesis. *LINK‐A* overexpressing MCF‐10A and control cells were stimulated with HB‐EGF, and the conditioned media was obtained to treat beige adipocytes (**Figure** **6**a). Notably, conditioned media of HB‐EGF‐stimulated MCF‐10A suppressed NE‐induced UCP1 protein expression in beige adipocytes, which was further enhanced by *LINK‐A* overexpression (Figure 6b). A similar phenomenon was observed for the expression levels of P‐PKA and its downstream P‐HSL in adipocytes (Figure 6b), and the mRNA levels of the heat production genes in adipocytes (Figure 6c–f). The reduction in adipocyte thermogenesis caused by the conditioned media of HB‐EGF‐stimulated *LINK‐A* overexpressed‐MCF‐10A cells was alleviated by the HIF1α knockdown (Figure 6b–f) or by the inhibitors of the HIF1α downstream cytokines (such as CXCL16 and IL‐1β) (Figure 6g–l). Together with the in vivo results that HFD‐KI mice exhibited low levels of energy expenditure (Figure 1p,r) and had poor adaptive thermogenesis under cold conditions (Figure 2b–d). These cellular experiments showed that the activated *LINK‐A*/HB‐EGF/HIF1α pathway in mammary cells could affect adipocyte thermogenesis likely by altering the inflammatory microenvironment of surrounding adipocytes (Figure 6m).

### 2.7 ASO Drug Inhibiting *LINK‐A* Attenuates Obesity and Metabolic Disorders in Mice

**Figure 7:**
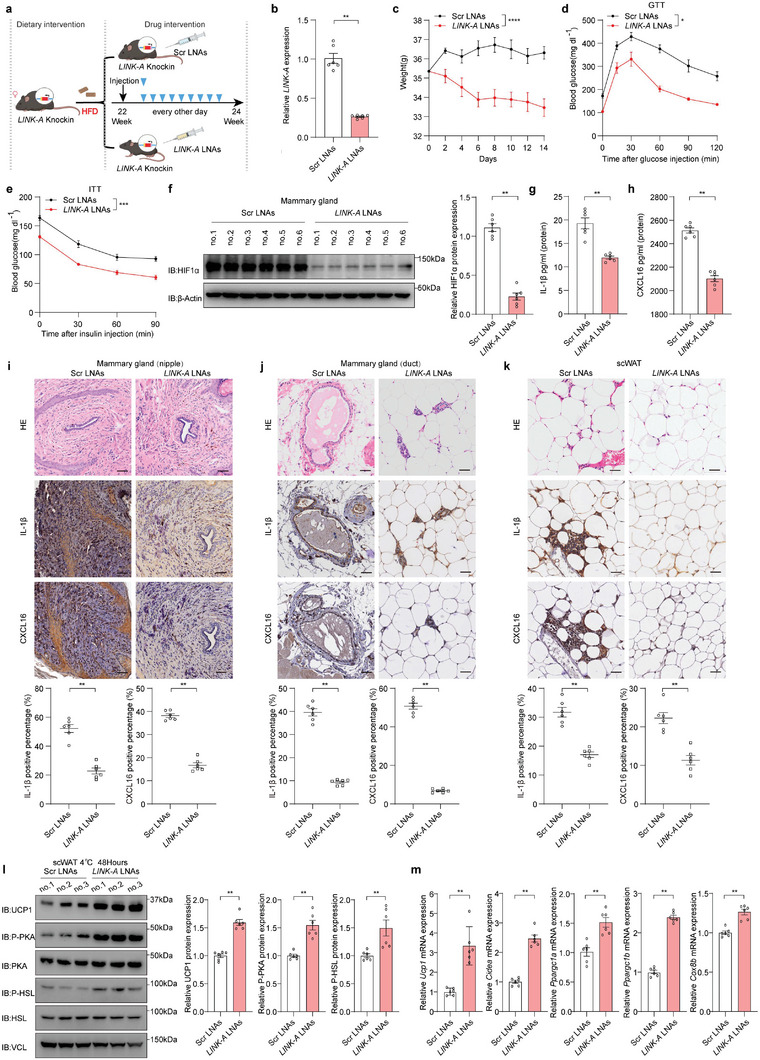
ASO drug inhibiting *LINK‐A* attenuates obesity and metabolic disorders in mice a) Schematic diagram of the mouse mammary gland in situ *LINK‐A* LNAs injection strategy. b) *LINK‐A* levels in mice mammary glands injected with *LINK‐A* LNAs in situ for two weeks by qRT‐PCR. Data presented as mean ± SEM, per group n = 6, Mann–Whitney U‐test, **p<0.01. c) Body weight changes in HFD‐KI mice with *LINK‐A* LNAs or Scr LNAs treatment. Data presented as mean ± SEM, per group n = 6, two‐way ANOVA, ns = no significance, ****p <0.0001. d,e) GTT(d) and ITT(e) of HFD‐KI mice with *LINK‐A* LNAs or Scr LNAs treatment. Data presented as mean ± SEM, per group n = 6, two‐way ANOVA, *p <0.05, ***p<0.001. f) The HIF1α protein levels in the mammary gland of HFD‐KI mice with *LINK‐A* LNAs or Scr LNAs treatment were detected by immunoblot analysis, and the HIF1α protein levels were quantified using ImageJ. Data presented as mean ± SEM, per group n = 6, Mann–Whitney U‐test, **p <0.01. g,h) The plasma concentrations of IL‐1β(g), and CXCL16(h) in HFD‐KI mice with *LINK‐A* LNAs or Scr LNAs treatment were measured by ELISA. Data presented as mean ± SEM, per group n = 6, Mann–Whitney U‐test, **p <0.01. i–k) Representative images of H&E‐stained sections and IHC of the mammary gland (nipple)(i), mammary gland (duct)(j), and scWAT(k), and the IL‐1β or CXCL16 protein levels were analyzed using ImageJ. Scale bar: 50 µm. Data presented as mean ± SEM, per group n = 6, Mann–Whitney U‐test, **p <0.01. l) HFD‐KI treated with *LINK‐A* LNAs or Scr LNAs for two weeks were placed at 4 °C, 48 h, and their protein levels of UCP1, P‐PKA, PKA, P‐HSL, HSL in scWAT were detected by immunoblot analysis, and the protein levels were quantified using ImageJ. Data presented as mean ± SEM, per group n = 6, Mann–Whitney U‐test, **p <0.01. m) HFD‐KI treated with *LINK‐A* LNAs or Scr LNAs for two weeks were placed at 4 °C, 48 h, and their mRNA levels of thermogenic genes in scWAT were detected by qRT‐PCR. Data presented as mean ± SEM, per group n = 6, Mann–Whitney U‐test, **p <0.01.

To investigate the effect of targeting *LINK‐A* in obesity and metabolic disorders in HFD‐KI mice, we designed five ASOs complementary to *LINK‐A*. We selected one to enhance its stability using locked nucleic acid (LNA) modification.^[^ ^60^ ^]^ The effectiveness of *LINK‐A* LNAs was demonstrated by the considerably decreased *LINK‐A* levels in MDA‐MB‐231 and MCF‐10A cells after treatment with different concentrations of *LINK‐A* LNAs (Figure S4a,b, Supporting information). We observed that *LINK‐A* LNAs resulted in decreased levels of HIF1α protein, mRNA levels of HIF1α downstream gene transcripts, *HB‐EGF*, and inflammatory factors but had no effect on mRNA levels of HIF1α (Figure S4c,d, Supporting information). Given that *LINK‐A* expression was the highest in mouse mammary glands (Figure 1e), we directly injected *LINK‐A* LNAs into the mammary glands of HFD‐KI mice to inhibit *LINK‐A* levels to the greatest extent (**Figure** **7**a). As expected, the level of *LINK‐A* in the mammary tissue of mice decreased by approximately 70% after 2 weeks of receiving *LINK‐A* LNAs in situ in the mammary glands (Figure 7b). Of note, *LINK‐A* LNAs treatment alleviated obesity and metabolic disorders in mice, as evidenced by weight loss, reduced fasting glucose, and increased insulin sensitivity (Figure 7c–e; Figure S4e, Supporting information). *LINK‐A* LNAs also inhibited the activated HIF1α signaling in the mouse mammary glands (Figure 7f), including the transcript levels of HIF1α downstream factors and inflammatory factors IL‐1β and CXCL16 (Figure S4f, Supporting information). Consequently, it reduced the concentrations of IL‐1β and CXCL16 in the plasma (Figure 7g,h) and improved insulin sensitivity (Figure 7e). IHC results showed that *LINK‐A* LNAs reduced the infiltration of IL‐1β and CXCL16 in the mammary tissue (Figure 7i,j) and the scWAT surrounding the mammary glands (Figure 7k). Mice housed in cold conditions (4 °C) showed increased thermogenic capacity of scWAT, including the induced expression of thermogenic proteins and genes (Figure 7l,m). In summary, inhibition of *LINK‐A* using *LINK‐A* LNAs effectively mitigated the obesity induced by HFD in *LINK‐A* KI mice by suppressing the activated *LINK‐A*/HB‐EGF/HIF1α loop.

### 2.8 The *LINK‐A* Expression was Positively Correlated with the Expression of Inflammatory Cytokines in Overweight People

**Figure 8:**
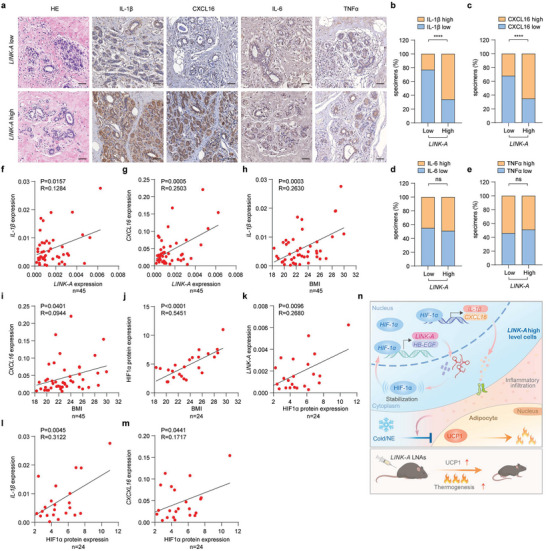
The *LINK‐A* expression was positively correlated with the expression of inflammatory cytokines in overweight people a–e) Representative images of H&E‐stained sections and IHC of breast tissue microarrays(a) of patients with overweight, and the inflammatory factor levels were analyzed using ImageJ. Scale bar: 50 µm. Data presented as mean ± SEM, n = 12, chi‐square test, ns = no significance, ****p <0.0001. f–i) Correlation between *IL‐1β*, *CXCL16*, *LINK‐A* and BMI. Data presented as mean ± SEM, n = 45, Pearson chi‐square test. *p <0.05, **p <0.01, ***p<0.001. j–m) Correlation between BMI, *IL‐1β*, *CXCL16*, *LINK‐A*, and HIF1α protein. Data presented as mean ± SEM, n = 24, Pearson chi‐square test. *p <0.05, **p <0.01, ***p <0.001, ****p <0.0001. n) Article General Mechanism Diagram.

We collected breast tissue microarrays to investigate the correlation between *LINK‐A* and inflammatory cytokines in individuals who are overweight (BMI≥ 25). We divided them into two groups based on the *LINK‐A* expression (Table S1, Supporting information). We found that the protein expression levels of Il‐1β and CXCL16 in breast tissues with high *LINK‐A* expression were considerably higher than those with low *LINK‐A* expression (**Figure** **8**a–c), whereas the expression of IL‐6 and TNFα remained unaltered (Figure 8a,d,e). Moreover, we found that both *IL‐1β* and *CXCL16* were positively correlated with *LINK‐A* and BMI, respectively (Figure 8f–i). Furthermore, a notable positive correlation between the HIF1α protein and the expression of *LINK‐A*, *IL‐1β*, and *CXCL16*, as well as BMI, was also observed (Figure 8j–m; Figure S5a, Supporting information).

In conclusion, our study showed that *LINK‐A* promoted HFD‐induced obesity by reducing thermogenesis through the HB‐EGF‐triggered stabilization of HIF1α. This directly induced activation of the *LINK‐A*/HB‐EGF/HIF1α loop, further altering the local adipose tissue microenvironment by releasing inflammatory factors IL‐1β and CXCL16. Based on in vivo experiments conducted in mice and the analysis of clinical samples, it is suggested that *LINK‐A* can be exploited as a valuable and efficacious therapeutic target for obesity induced by HFD (Figure 8n).

## 3 Discussion

The roles of lncRNAs in regulating the inflammatory microenvironment during the development of HFD‐induced obesity remain largely unknown. We constructed knock‐in mice overexpressing the obesity‐associated human lncRNA *LINK‐A*. We found that under HFD conditions, overexpression of *LINK‐A* impaired the adaptive thermogenesis in mice under cold conditions, which is one of the essential contributors to energy expenditure.^[^ ^53^ ^]^ By examining the levels of inflammatory factors, we found that elevated expression of *LINK‐A* promoted the expression and release of inflammatory factors IL‐1β and CXCL16. Further, co‐treatment of beige adipocytes with IL‐1β and CXCL16 factors suppressed the NE‐induced thermogenic capacity of beige. In conclusion, our data suggest that the remodeled inflammatory microenvironment in mammary epithelial cells may be a potential cause of *LINK‐A*‐induced obesity.

The specific molecular roles of lncRNAs in mammary epithelial cells regulating the inflammatory microenvironment remain unclear. We found that IL‐1β and CXCL16 are regulated by the transcription factor HIF1α. The protein and downstream signaling pathways of HIF1α were activated in HFD‐KI mice. Our previous study showed that *LINK‐A* and HB‐EGF together promoted the activation of the HIF1α signaling pathway in breast cancer cells.^[^ ^48^ ^]^ Furthermore, we found that HFD feeding increased HB‐EGF in mouse mammary tissue. We found that *LINK‐A* overexpressing MCF‐10A cells with HB‐EGF stimulation considerably stabilized HIF1α protein and induced IL‐1β and CXCL16 cytokines. *LINK‐A* and HB‐EGF were also positively regulated by HIF1α at the transcriptional level. Therefore, lncRNAs regulate the inflammatory microenvironment by forming a positive feedback loop between *LINK‐A* and HB‐EGF/HIF1α to amplify the transcription factor HIF1α signaling cascade, including its direct targets, such as IL‐1β and CXCL16.

Despite genome‐wide identification of lncRNAs in human diseases, the evidence suggesting the biological importance of lncRNAs in promoting the development and progression of obesity and metabolic syndrome is limited. Increased expression of *LINK‐A* in mouse mammary glands accelerates HFD‐induced obesity, suggesting its role in regulating body metabolism. In this study, targeting *LINK‐A* with the nucleic acid drug ASO could alleviate obesity and metabolic disorders in mice and further restore adipose tissue thermogenic capacity by remodeling the local microenvironment. Furthermore, clinical analysis has revealed that *LINK‐A* levels in female breast tissue are positively correlated with BMI and that LINK‐A and inflammatory factors (IL‐1β and CXCL16) are co‐expressed in the breast tissue of females who are overweight. Thus, human‐specific lncRNA *LINK‐A* KI transgenic mouse models are valuable model systems for studying human obesity and metabolic disorders.

Studies have shown that different tissues have different metabolic characteristics.^[^ ^61^, ^62^, ^63^ ^]^ For example, adipose tissue remodels rapidly in response to external signals to maintain the body's energy homeostasis through metabolic processes such as increased lipid storage or thermogenesis.^[^ ^61^ ^]^ In addition, increased metabolic rates in breast tissue are often thought to occur from pregnancy to lactation to support mammary gland growth, lactation, and breastfeeding.^[^ ^62^ ^]^ Therefore, overexpression *LINK‐A* in other tissues may have different mechanisms to affect metabolic diseases.

In summary, we generated a *de novo* KI mouse model to demonstrate *LINK‐A* to be an effective RNA‐based therapeutic drug target to treat HFD‐induced obesity, evidenced by the nucleic acid drug ASO preventing the developing HFD‐induced obesity in *LINK‐A* KI mice. We also used in vitro experiments to further reveal that under the regulation of the *LINK‐A*/HB‐EGF/HIF1α loop pathway, lncRNAs (such as *LINK‐A*) remodel the inflammatory microenvironment by inducing the expression of IL‐1β and CXCL16, thereby decreasing the surrounding adipocytes' thermogenic capacity, which seems to be crucial for promoting HFD‐induced obesity. In the future, we intend to focus on elucidating the mechanisms by which inflammatory factors IL‐1β and CXCL16 contribute to the development of obesity in HFD‐KI mice.

## 4 Experimental Section

### Cells Lines

Human mammary epithelial cell line MCF‐10A (cat.no.GNHu50), Human breast cancer cell line MDA‐MB‐231 (cat.no.TCHu227), human bone marrow mesenchymal stem cells (cat.no.SCSP‐450) and HEK‐293T (cat.no.GNHu44) were purchased from the National Collection of Authenticated Cell Cultures (China). A Dulbecco‐modified essential medium (DMEM) supplemented with 10% fetal bovine serum (FBS) was used for the maintenance of these cell lines at 37 °C in 5% CO_2_(v/v). STR profiling performed by vendors did not reveal any of the cell lines used in this study in the ICLAC or NCBI Biosample databases of commonly misidentified cell lines.

### Tissue Samples

A total of 45 breast tissues (>5 cm from the tumor) were obtained from patients who underwent surgery at the Huzhou First People's Hospital (Huzhou, China). The study protocol was approved by the Institutional Review Board of the Huzhou First People's Hospital (2021kyII055). All procedures were performed under the internal review and approval of the ethics committee of the Huzhou First People's Hospital. Participants were recruited from the Huzhou First People's Hospital without apparent bias, and all eligible participants were invited to participate. An informed consent policy was followed when collecting tissue samples. A total of 20 scWAT and VAT tissues (scWAT tissues were taken from the abdominal wall, and VAT tissues were taken from the appendices epiploicae) were obtained from patients who underwent surgery at the Affiliated Hangzhou First People's Hospital, Zhejiang University School of Medicine (Hangzhou, China). The study protocol was approved by the Institutional Review Board of the Affiliated Hangzhou First People's Hospital, Zhejiang University School of Medicine (ZN‐20230918‐0211‐02). All procedures were performed under the internal review and approval of the ethics committee of the Affiliated Hangzhou First People's Hospital, Zhejiang University School of Medicine. Participants were recruited from the Affiliated Hangzhou First People's Hospital, Zhejiang University School of Medicine without apparent bias, and all eligible participants were invited to participate. An informed consent policy was followed when collecting tissue samples. Body mass index (BMI) is defined as body mass in kilograms divided by height in meters squared (kg m^−2^)^[^ ^46^ ^]^ The World Health Organization (WHO) classifies adults with a BMI <18.5 kg m^−2^ as “underweight,” adults with an 18.5 ≤ BMI <25 kg m^−2^ as “normal,” and adults with a BM≥ 25 kg m^−2^ as “overweight”.^[^ ^47^ ^]^ 65 patient samples were grouped according to BMI. In Table S1 (Supporting Information), we provide detailed clinical information.

### Mice

Human *LINK‐A* Knock in TG mice was constructed by Cyagen Biosciences Inc. (Guangzhou, China). Genotype identification PCR primers are shown in Table S3 (Supporting Information). Mice were reared normally until eight weeks, and then female (C57BL/6 and *LINK‐A* KI) mice were grouped for ND or HFD for 12 weeks, and subsequent experiments were performed at postnatal week 20. All animal experiments were performed according to protocols approved by the Institutional Animal Care and Use Committee. The care of the experimental animals followed the Zhejiang University Laboratory Animal Committee guidelines and was approved (ZJU20220101).

### Antibodies

Table S2 (Supporting Information) provides detailed information about the reagents and materials used.

### Mice Housing

Six mice were housed in each cage and maintained in SPF‐level environments with temperature (22 ± 1 °C) and humidity (60% ± 10%), 12 h light/12 h dark cycles throughout the study, and were provided with ND (1 010 063, Xietong Shengwu) or HFD (D12492i, Research Diets) and ultrapure water, except for fasting experiments. All experiments were performed using female mice, genotypes, and the figures in the figure legends unless otherwise stated.

### Obesity‐Associated LncRNA Screening

By analyzing RNA‐seq data (GSE111358) from a chronic inflammatory disease: hip osteoarthritis, 198 lncRNAs were found to be significantly differentially expressed (FDR ≤ 0.05) between normal tissue and normal cartilage.^[^ ^43^ ^]^ In addition to this, 557 and 748 abnormally expressed lncRNAs were identified in the cardiovascular disease: GSV varicose veins (GSE51260) and autoimmune disease: rheumatoid arthritis (GSE181614) datasets, respectively, in the microarray dataset analysis.^[^ ^44^, ^45^ ^]^ Finally, it was found that *LINK‐A* was present in the differentially expressed LncRNAs of all three disease groups.

### *LINK‐*A Homology Analysis

The Blast method was first utilized to find the conserved sequences of *LINK‐A* in different species, and multiple sequence comparison work was performed using FastTree (http://meta.microbesonline.org/fasttree/). The sequence Logos method in the ggmsa package (R version 4.3.0) was then used to visually identify sequence patterns in the Multiple Sequence Alignment (MSA).

### Cloning Procedures, Cell Transfection, and Lentiviral‐Based Gene Transduction

Various plasmids required for the experiments were constructed by PCR using pLVX‐puro, pCDH‐puro, or pGL4 vectors. For detailed sequences, see Tables S3 and S4 (Supporting Information). Short interfering RNA and plasmid transfections were performed using Liposomal Transfection Reagent (YEASEN). Lentivirus was generated in HEK‐293T cells using the packaging vectors VSVG and psPAX2. Viruses were harvested 48 and 72 h after transfection and transfected with HEK‐293T, MCF‐10A cells followed by 1 µg mL^−1^ puromycin selection. In the functional studies, *LINK‐A* overexpression or HIF1α knockdown cells were verified by qRT‐PCR.

### Induced Differentiation of Beige Fat^[^ ^64^, ^65^ ^]^

Human bone marrow mesenchymal stem cells (BMSCs) were grown in DMEM supplemented with 10% FBS, 20 mm HEPES pH 7.2, and 1% Pen/Strep at 37 °C and 5% CO2 in 12‐well plates. The cells were grown in culture dishes, and after they reached 90% confluence, the medium was replaced (DMEM high glucose, supplemented with 10% FBS and 1% Pen/Strep) and the cells were cultured for two days. Two days later the cultures were incubated with 1 mL of “differentiation medium” for 2 days. The differentiation medium was formulated by adding insulin (20 nm), dexamethasone (1 µm), IBMX (0.5 mm), T3 (1 nm), indomethacin (0.125 mm), and rosiglitazone (2.8 µm) to the original. The medium was discarded on day 4 and incubated for 2 days with a new “differentiation medium” prepared by adding insulin (20 nm), T3 (1 nm), and rosiglitazone (2.8 µm) to the stock solution. The medium was discarded on day 6 and incubated for 2 days with a new “differentiation medium” prepared by adding insulin (20 nm) and rosiglitazone (2.8 µm) to the stock solution. On day 8 cell differentiation was completed and subsequent experiments could be performed.

### Adipocyte Thermogenic Capacity Experiment

It was shown that NE stimulates thermogenesis in brown and beige adipocytes by activating β3‐adrenergic signaling, to investigate the thermogenic capacity of adipocytes.^[^ ^66^ ^66]^ Beige fat with NE (Supelco) was treated as a thermogenic inducer and examined the thermogenic capacity of adipocytes.

### IL‐1β Inhibitor or/and CXCL16 Inhibitor Treated Adipocytes

After successful induction of beige adipocytes, soluble IL‐1β inhibitor (VX‐765, MCE, 10 µm) or/and soluble CXCL16 inhibitor (GI254032, Selleck, 3 µm) was added to the medium, and cells were cultured for 24 h, followed by collecting cells for subsequent experiments.

### Quantitative IL‐1β and CXCL16 Measurement

The concentrations of IL‐1β and CXCL16 in MCF‐10A cell supernatants after treatment with different conditions were measured using the Human IL‐1β ELISA kit (absin, abs159986) and the Human CXCL16 ELISA kit (R&D Systems, DCX160). The concentrations of IL‐1β and CXCL16 in mouse plasma were measured using the Mouse IL‐1β ELISA kit (absin, abs520001) and the Mouse CXCL16 ELISA kit (R&D Systems, DY503). Specific experimental procedures were performed according to the reagent supplier's instructions.

### ChIP‐qPCR Assay

The cell line used in this experiment was the mammary epithelial cell line MCF‐10A. First, the fixative was prepared, and formaldehyde (1%) and protease inhibitor were added to the cell culture medium and then added to the MCF‐10A cells. after 5 min, glycine (125 mm) was added and mixed. after 3 min, the liquid was discarded, and the cells were collected after washing three times with pre‐chilled 1×PBS. After resuspension of the cells using PBS, the cells were treated with ultrasound to form a 100–500 bp DNA fragment. Next, protein A/G PLUS‐agarose (Santa Cruz, sc‐2003) was added with HIF1α antibody, mixed lighting, and incubated at 4 °C with rotation. After incubation, the beads were washed using a washing solution. The RNA and protein on the magnetic beads were removed using SDS and proteinase K. Finally, DNA was extracted using the DNA purification kit, and the obtained DNA was tested for enrichment ploidy using qPCR. The specific primer information is shown in Table S3 (Supporting Information).

### Quantitative RT‐PCR Analysis

The tissue (need to be ground with magnetic beads in advance) or cells to be analyzed by quantitative RT‐PCR were collected and added to TRIzol (Invitrogen), mixed thoroughly, and then added 1/5 volume of trichloromethane, shaken and mixed vigorously, and then centrifuged at the highest speed for 10 min (4 °C), the supernatant was taken and an equal volume of isopropanol was added, mixed lightly (6–8 times) and then left for 10 min, the highest rpm centrifugation for 10 min (4 °C), discard the supernatant, wash twice using 75% ethanol, and dissolve with DEPC water to obtain RNA solution. The extracted RNA was reverse transcribed into cDNA using 5 × HiScript II qRT SuperMix (R222‐01, Vazyme), and then the qRT‐PCR reaction system was configured using SYBR qPCR Master Mix (Q711‐012, Vazyme), and the reaction system was performed on a CFX ConnectTM Real‐Time System (Thermo Fisher Scientific). The results obtained were used to calculate the relative mRNA expression using the 2(‐ΔΔCT) method. Detailed primer sequences are summarized in Table S3 (Supporting Information).

### Dual‐Luciferase Reporter Assay

First, pGL4‐*LINK‐A*, pGL4‐HB‐EGF, pGL4‐IL‐β, pGL4‐CXCL16, pGL4‐Vector, their mutants, were co‐transfected with pLVX‐HIF1α into HEK‐293T cells. Cells were collected after 48 h. Detection was performed using the Dual‐Luciferase Reporter Gene Assay Kit (YEASEN). Results were read using a Multi‐Detection Microplate Reader (Bio‐Tek). The final results were normalized to Renilla luciferase activity. These results are representative of three independent experiments.

### Glucose and Insulin Tolerance Tests

According to different experimental needs, mice for glucose and insulin tolerance indexes were tested. GTT refers to the mouse glucose tolerance test and ITT refers to the mouse insulin tolerance test. Mice were injected with 40% glucose solution (1 g kg^−1^) after 16 h of fasting, and the blood glucose levels at 0, 15, 30, 60, 90, and 120 min after injection were measured as mouse GTT. Mice were injected with insulin (Novolin 30R, 0.25 IU/kg) after 6 h of fasting, and the blood glucose levels at 0, 15, 30, 60, and 90 min after injection were measured as mouse ITT. ITT in mice. blood glucose levels were measured with a blood glucose meter (YuWell).

### Energy expenditure

VO_2_, energy expenditure, and RER were quantified in mice at 20 weeks using CLAMS (Columbus Instruments).

### Cold Challenge Experiment

Mice were kept at 30 °C and fasted for 16 h, then transferred to pre‐cooled CLAMS(Columbus Instruments) at 4 °C, where mice were not fed and body temperature, VO_2_, energy expenditure, and RER were monitored hourly for 6 h. The mice were euthanized at the end of the experiment.

### Mice Treatment Procedures

Mice were injected intraperitoneally with *LINK‐A* LNA or Scr LNA (5 mg kg^−1^, RIBOBIO, every other day). The sequence of *LINK‐* *A* LNA and Scr LNA are shown in Table S3 (Supporting Information). GTT, ITT, and thermogenesis were measured 2 weeks after administration. Tissues were collected at the end of all experiments. All animal experiments were performed according to protocols approved by the Institutional Animal Care and Use Committee. The care of the experimental animals was following the guidelines of the Zhejiang University Laboratory Animal Committee and was approved.

### Histological Analysis

The immunohistochemical procedures of paraffin‐embedded tissue sections were as follows: xylene dewaxing; gradient ethanol rehydration; section antigen repair; primary antibody incubation; secondary antibody incubation; hematoxylin staining; gradient ethanol dehydration; xylene fixation; and finally the image was obtained by Olympus DP72 microscope. The positive rate was quantified by ImageJ. The total protein expression was calculated according to the percentage and intensity of positive cells. The average scores of all samples were used as cut‐off points to define high and low protein expression. The correlation between indexes and BMI was analyzed using Spearman rank correlation.

### Immunoblotting

The tissue (that needs to be ground in advance) and cell samples were cracked by adding 1 × RIPA buffer. The RIPA buffer, consisting of Tris‐HCl pH 7.5 (50 mm); NaCl (450 mm); NP‐40 (1%); SDS (0.1%); deoxycholic (0.5%) and protease inhibitors, was cleaved on ice for 30 min and shaken every 10 min. Then centrifuge at maximum speed for 10 min (4 °C). The collected supernatant was the protein solution, which was quantified by BCA (P0009, Beyotime). Then add 5 × loading to the protein solution and denature for 10 min (100 °C). The 5 × loading included (Tris‐HCl pH 6.8 (250 mm); SDS (10%); BPB (0.5%); glycerol (50%) and β‐mercaptoethanol (5%). The protein was added to the SDS‐PAGE gel, and the protein was separated in the gel by applying voltage. Transfer the protein from the gel to the PVDF membrane. Signals were detected by ChemiDocTMTouch Imaging System (BIO‐RAD) chemiluminescent imaging system using SuperKine West Femto Maximum Sensitivity Substrate.

### Statistical Analysis and Reproducibility

In each experiment, group, and condition, 3–6 mice/cells/replicates were used. For western blotting, the representative images are shown. Each of these experiments was independently repeated 3–6 times. Relative quantities of gene expression levels were normalized to β‐actin/GAPDH/18S/36B4. Results are reported as mean ± Standard Error of Mean (SEM) of at least three independent experiments. Comparisons were performed using unpaired Student's t‐test, Mann–Whitney U‐test, one‐way ANOVA, two‐way ANOVA, or chi‐square test, as indicated in individual Figures. Prism Software (GraphPad, La Jolla, CA). The experiments were not randomized. The investigators were not blinded to allocation during experiments and outcome assessment.

### Ethical Statement

All research involving human participants was conducted under all applicable ethical regulations. The First People's Hospital of Huzhou (Zhejiang, China) collected breast tissue samples from patients. Huzhou First People's Hospital's Institutional Review Board approved the study protocol for the clinical organization (2021kyII055). Participants were recruited without bias from Huzhou First People's Hospital and written informed consent was obtained from all participants participating in the study. The Affiliated Hangzhou First People's Hospital, Zhejiang University School of Medicine (Zhejiang, China) collected scWAT and VAT tissue samples from patients. Affiliated Hangzhou First People's Hospital, Zhejiang University School of Medicine's Institutional Review Board approved the study protocol for the clinical organization (ZN‐20230918‐0211‐02). Participants were recruited without bias from Affiliated Hangzhou First People's Hospital, Zhejiang University School of Medicine and written informed consent was obtained from all participants participating in the study. Institutional Animal Care and Use Committee (IACUC)‐approved protocols were followed for all animal experiments (ZJU20220101). Upon approval of the Zhejiang University Laboratory Animal Committee, experimental animals were cared for according to its guidelines.

## Conflict of Interest

The authors declare no conflict of interest.

## Author Contributions

Y.C., H.C., and Y.W. contributed equally to this work. A.L. and J.L. conceived and designed the research. Y.C., H.C., and Y.W. performed most of the biochemical and molecular experiments and bioinformatics analysis, with assistance from F.L., X.F., C.S., X.S., M.T., K. L., L.Q., J.Y., Y.C., H.C., and Y.W. performed in vivo experiments. X.S. performed bioinformatics analysis. Y.Y. and B.L. contributed to the clinical sample collection. S.X., Q.S., D.N., W.L., Q.Y., X.W., and J.S. contributed to the discussion and data interpretation. A.L., J.L., and Z.Y. edited the manuscript. A.L., J.L., and Y.C. wrote the manuscript.

## Supporting information

Supporting Information

## Data Availability

The data that support the findings of this study are available in the supplementary material of this article.
