# Supplementary material for: LncRNA LINK‐A Remodels Tissue Inflammatory Microenvironments to Promote Obesity
Source: Adv Sci (Weinh). 2023 Dec 25;11(10):2303341. doi: 10.1002/advs.202303341 (PMC10933663; doi:10.1002/advs.202303341)
Supplement: Supplementary file 1 — Supporting Information [file ADVS-11-2303341-s001.pdf]

ADVANCED SCIENCE
Open Access

Supporting Information

for *Adv. Sci.*, DOI 10.1002/advs.202303341

LncRNA *LINK*-A Remodels Tissue Inflammatory Microenvironments to Promote Obesity

Yu Chen, Hui Chen, Ying Wang, Fangzhou Liu, Xiao Fan, Chengyu Shi, Xinwan Su, Manman Tan, Yebin Yang, Bangxing Lin, Kai Lei, Lei Qu, Jiecheng Yang, Zhipeng Zhu, Zengzhuang Yuan, Shanshan Xie, Qinming Sun, Dante Neculai, Wei Liu, Qingfeng Yan, Xiang Wang, Jianzhong Shao, Jian Liu and Aifu Lin**

Supporting Information

LncRNA *LINK-A* Remodels Tissue Inflammatory Microenvironments to Promote Obesity

Yu Chen, Hui Chen, Ying Wang, Fangzhou Liu, Xiao Fan, Chengyu Shi, Xinwan Su, Manman Tan, Yebin Yang, Bangxing Lin, Kai Lei, Lei Qu, Jiecheng Yang, Zhipeng Zhu, Zengzhuang Yuan, Shanshan Xie, Qinming Sun, Dante Neculai, Wei Liu, Qingfeng Yan, Xiang Wang, Jianzhong Shao, Jian Liu and Aifu Lin**

Figures S1 to S5

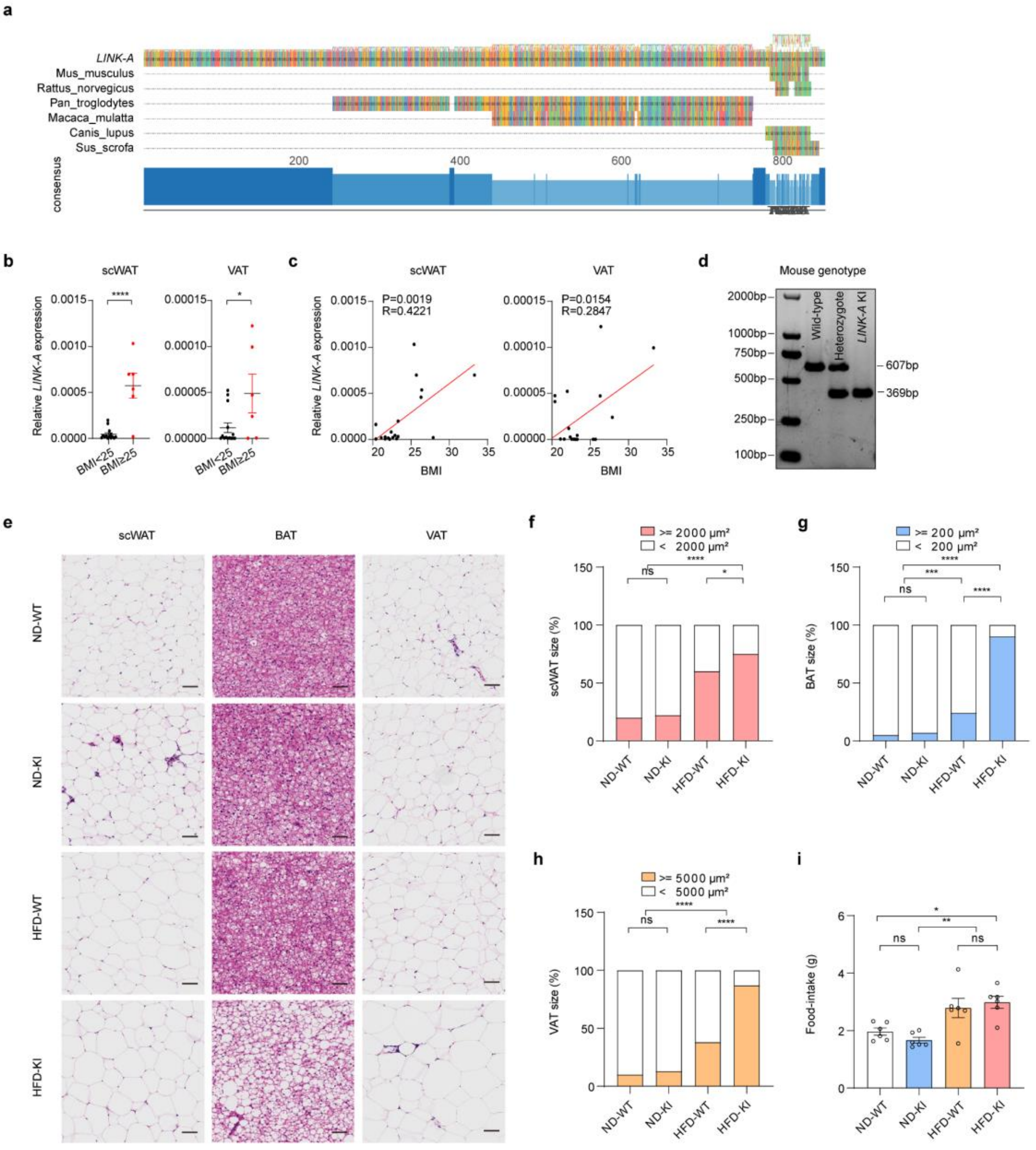

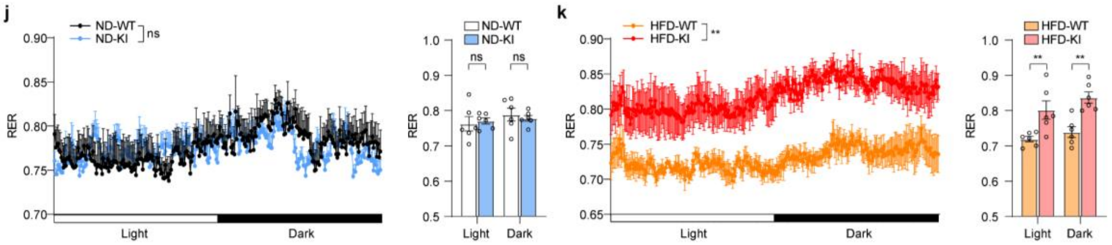

Figure S1. ***LINK-A*** **overexpression in mice promotes HFD-induced obesity a)** The stacked graph for *LINK-A* conserved regions of sequences in various species. To be specific, the sequence of MSA is depicted as an uninterrupted, semi-opaque line. Each line represents a sequence. A: red, C: blue, T: green, G: yellow. The Sequence logo visualization method calculates the frequency of characters in each column in the MSA, and scales and stacks the characters in each column according to the frequency. **b)** *LINK-A* expression was assessed by qRT-PCR in scWAT(left) and VAT(right) tissues of patients in the BMI < 25 and BMI ⩾ 25 groups. Data presented as mean ± SEM, n=14, 6, Student's t-test, the data conform to normal or lognormal distribution, **p*<0.05, *****p*<0.0001. **c)** Correlation analysis of *LINK-A* expression in the in scWAT(left) and VAT(right) tissues of clinical patients with patient BMI. Data presented as mean ± SEM, n=20, Pearson chi-square test, *p*=0.0019(scWAT), *p*=0.0154(VAT). **d)** Identification of mouse genotypes by pulsed electric field gel electrophoresis. **e–h)** Representative images of H&E-stained sections of different adipose tissues(e). The distribution of adipocyte size of scWAT(f), BAT(g), and VAT(h) of ND-WT, ND-KI, HFD-WT, and HFD-KI mice were analyzed using ImageJ. Scale bar: 50μm. Data presented as mean ± SEM, per group n=6, chi-square test, ns=no significance, **p*<0.05, ****p*<0.001, *****p*<0.0001. **i)** Food intake of WT or *LINK-A* KI mice fed ND or HFD. Data presented as mean ± SEM, per group n=6, one-way ANOVA, ns=no significance, **p*<0.05, ***p*<0.01. **j–k)** Line graphs(left) and bar charts(right) of RER in WT and *LINK-A* KI mice fed ND(j) or HFD(k). Data presented as mean ± SEM, per group n=6, two-way ANOVA, ns=no significance, ***p*<0.01.

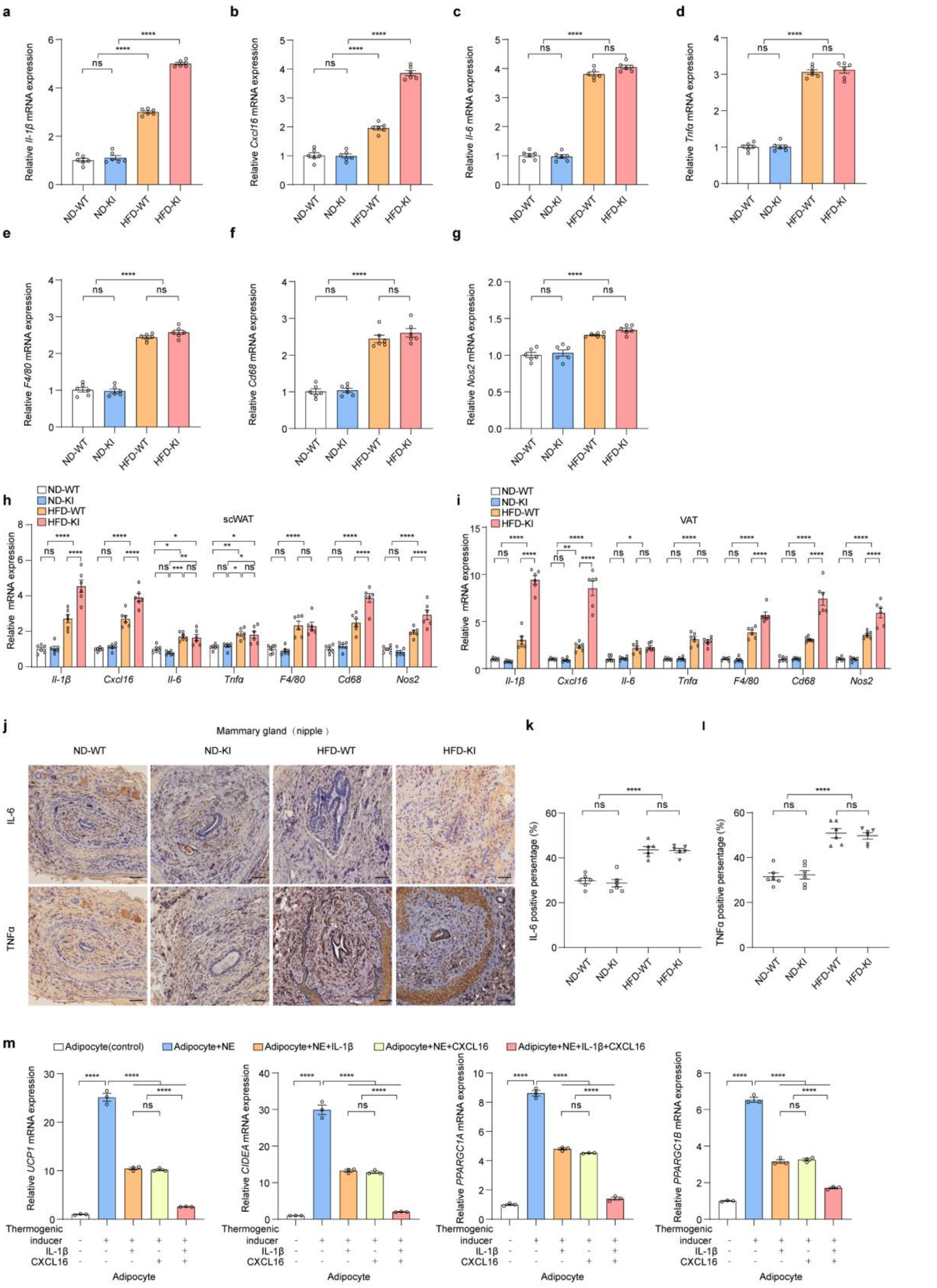

Figure S2. ***LINK-A*** **overexpression reduces adaptive thermogenesis in HFD-fed mice by remodeling the regional inflammatory microenvironment a–g)** Inflammatory factor mRNA expression levels in the mammary glands of ND-WT, ND-KI, HFD-WT, and HFD-KI mice were detected by qRT-PCR. Data presented as mean ± SEM, per group n=6, one-way ANOVA,

ns=no significance, ****$p<0.0001$. **h–i)** Inflammatory factor mRNA expression levels in the scWAT(h) and VAT(i) of ND-WT, ND-KI, HFD-WT, and HFD-KI mice were detected by qRT-PCR. Data presented as mean ± SEM, per group n=6, two-way ANOVA, ns=no significance, *$p<0.05$, **$p<0.01$, ***$p<0.001$, ****$p<0.0001$. **j–l)** Representative images of IHC of mammary glands (nipple)(j). The IL-6(k) or TNFα(l) levels were analyzed using ImageJ. Scale bar: 50μm. Data presented as mean ± SEM, per group n=6, one-way ANOVA, ns=no significance, ****$p<0.0001$. **m)** The thermogenic genes mRNA levels in adipocytes treatment using IL-β or/and CXCL16 cytokines by qRT-PCR. Data presented as mean ± SEM, pooled data from three independent experiments, one-way ANOVA, ns=no significance, ****$p<0.0001$.

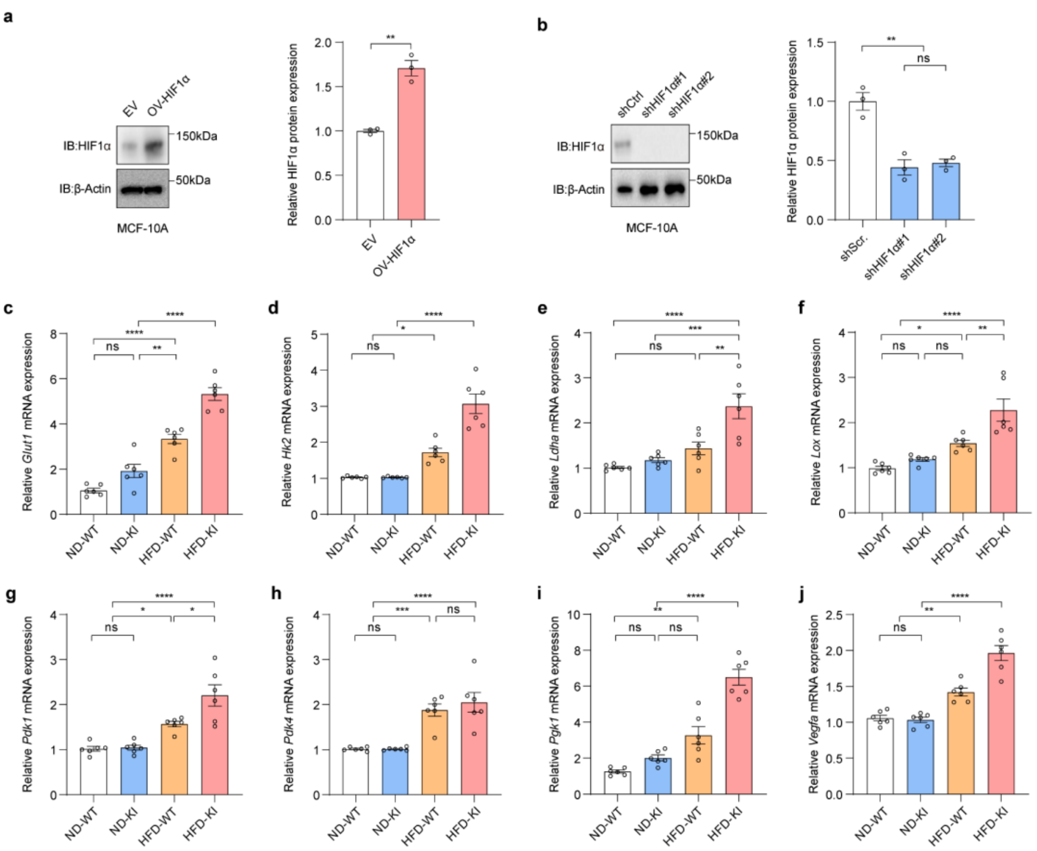

Figure S3. ***LINK-A*** **overexpression induces inflammatory factor expression by stabilizing HIF1α through the HFD-induced HB-EGF a)** The HIF1α protein levels in MCF-10A EV or HIF1α overexpression cells were detected by immunoblot analysis, and the HIF1α protein levels were quantified using ImageJ. Data presented as mean ± SEM, pooled data from three independent experiments, unpaired t-test, **$p<0.01$. **b)** The HIF1α protein levels in MCF-10A shControl (shCtrl) or shHIF1α (shHIF1α#1, shHIF1α#2) cells by immunoblot analysis, and the

HIF1α protein levels were quantified using ImageJ. Data presented as mean ± SEM, pooled data from three independent experiments, one-way ANOVA, ns=no significance, **p<0.01. **c–j)** The HIF1α downstream target genes mRNA expression in the mammary glands of ND-WT, ND-KI, HFD-WT, and HFD-KI mice were detected by qRT-PCR. Data presented as mean ± SEM, per group n=6, one-way ANOVA, ns=no significance, *p<0.05, **p<0.01, ****p<0.0001.

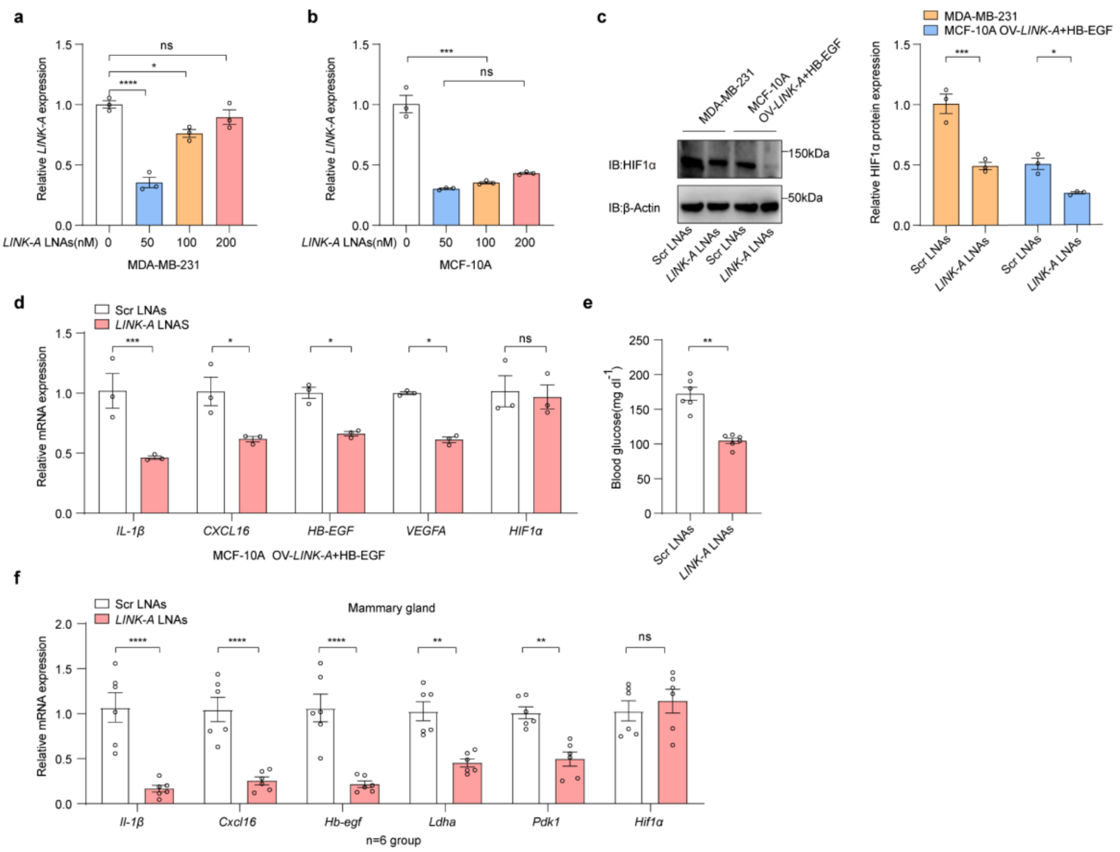

Figure S4. ASO drug inhibiting *LINK-A* attenuates obesity and metabolic disorders in mice a-b) The *LINK-A* levels in the MDA-MB-231(a) and MCF-10A(b) treated with different concentrations of *LINK-A* LNAs were detected by qRT-PCR. Data presented as mean ± SEM, pooled data from three independent experiments, one-way ANOVA, ns=no significance, *p<0.05, ***p<0.001, ****p<0.0001. **c)** The HIF1α protein levels in the MDA-MB-231 and MCF-10A with or without *LINK-A* LNAs treatment were detected by immunoblot analysis, and the HIF1α protein levels were quantified using ImageJ. Data presented as mean ± SEM, pooled data from three independent experiments, two-way ANOVA, *p<0.05, ***p<0.001. **d)** The mRNA expressions of *HIF1α* or HIF1α downstream genes were detected by qRT-PCR. Data presented as mean ± SEM, pooled data from three independent experiments, two-way ANOVA,

ns=no significance, **p*<0.05, ****p*<0.001. **e)** The fasting glucose in HFD-KI mice with *LINK-A* LNAs or Scr LNAs treatment. Data presented as mean ± SEM, per group n=6, Mann-Whitney U-test, ***p*<0.01. **f)** The mRNA levels in the mammary gland of HFD-KI mice with *LINK-A* LNAs or Scr LNAs treatment for two weeks were detected by qRT-PCR. Data presented as mean ± SEM, per group n=6, two-way ANOVA, ns=no significance, ***p*<0.01, *****p*<0.001.

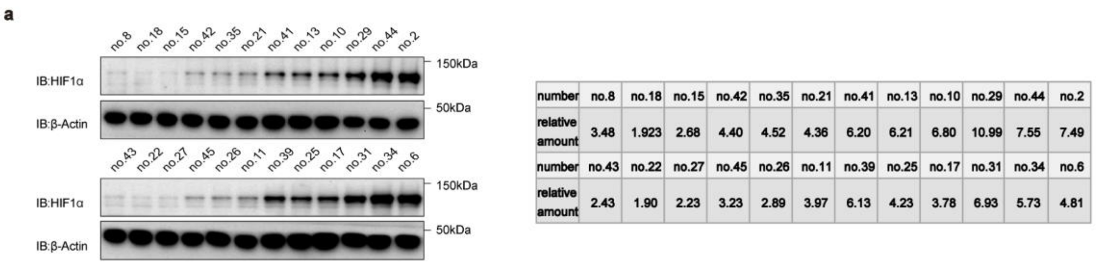

Figure S5. The expression levels of HIF1α protein in clinical breast samples. a) The HIF1α protein level in the breast tissue of clinical patients was detected by immunoblot analysis, and the HIF1α protein was quantified using ImageJ. n=24.

Table S1 to S4

Table S1. Clinicopathological Parameters of Tissue Microarrays Used in the Study

**Table S1.Clinicopathological Parameters of Tissue Microarrays Used in the Study**									
**Breast Tissue Microarrays Used in the Study**									
number	Age	Sex	Height (m)	Weight (kg)	BMI	*LINK-A* expression	*IL-1β* mRNA expression	*CXCL16* mRNA expression	HIF1α Protein level
1	50-60	F	1.57	55	22.31328	0.001022	0.0037399	0.0109827	N/A
2	40-50	F	1.61	66	25.46198	0.000913	0.0031317	0.0537407	7.49
3	20-30	F	1.64	61	22.67995	0.000284	0.0060806	0.0297091	N/A
4	40-50	F	1.58	49	19.62826	0.000168	0.0012892	0.0073193	N/A
5	40-50	F	1.59	50	19.7777	0.002592	0.0014706	0.0155814	N/A
6	50-60	F	1.6	55	21.48438	0.000436	0.0009997	0.0312578	4.816427
7	50-60	F	1.54	57	24.03441	0.004735	0.000963	0.220718	N/A
8	40-50	F	1.52	60	25.96953	0.000448	0.0024108	0.0109425	3.48
9	40-50	F	1.63	59	22.20633	0.000576	0.0169038	0.0176296	N/A
10	60-70	F	1.54	64	26.986	0.001717	0.0025334	0.0155565	6.8
11	40-50	F	1.52	47	20.3428	0.000457	0.0074517	0.0826515	3.97707
12	60-70	F	1.55	52	21.64412	0.002324	0.0007774	0.0181257	N/A
13	40-50	F	1.58	70	28.04038	0.005255	0.0100917	0.10785	6.213457
14	50-60	F	1.58	50	20.02884	0.00096	0.0004588	0.0074244	N/A
15	40-50	F	1.61	55	21.21832	0.000362	0.0022103	0.0862659	2.684295
16	50-60	F	1.6	55	21.48438	0.001749	0.0091718	0.1676537	N/A
17	60-70	F	1.58	52.5	21.03028	0.001136	0.0002348	0.0267928	3.783127
18	40-50	F	1.57	50	20.2848	0.001121	0.008899	0.0073349	1.923465
19	40-50	F	1.58	65	26.03749	0.000845	0.0051617	0.038061	N/A

20	30-40	F	1.55	72	29.96878	0.004556	0.0110747	0.0606632	N/A
21	60-70	F	1.58	63.5	25.43663	0.003113	0.0044138	0.07480802	4.36
22	30-40	F	1.56	49	20.13478	0.00096	0.0021717	0.0391797	1.901839
23	40-50	F	1.5	54	24	0.000664	0.0048995	0.0348943	N/A
24	40-50	F	1.67	63	22.58955	0.000457	0.0004594	0.0165293	N/A
25	40-50	F	1.59	48	18.98659	0.00078	0.0026087	0.0114104	4.234577
26	30-40	F	1.59	57	22.54658	0.003225	0.0038366	0.00423	2.891804
27	50-60	F	1.63	62	23.33547	0.002305	0.0030872	0.0187445	2.234565
28	50-60	F	1.58	54	21.63115	0.000348	0.0028461	0.0177258	N/A
29	50-60	F	1.53	69	29.47584	0.0062549	0.027552	0.154281	10.99793
30	50-60	F	1.55	45	18.73049	0.000549	0.0033284	0.0303122	N/A
31	50-60	F	1.63	76	28.60476	0.003585	0.0190846	0.0215266	6.937737
32	30-40	F	1.62	52	19.81405	0.001378	0.0081364	0.035292	N/A
33	40-50	F	1.59	58	22.94213	0.002143	0.0033893	0.0658015	N/A
34	40-50	F	1.65	65	23.87511	0.000614	0.0032358	0.0470298	5.739
35	30-40	F	1.56	56	23.01118	0.000501	0.0127047	0.0249603	4.523458
36	30-40	F	1.61	66	25.46198	0.000688	0.0083113	0.0731988	N/A
37	40-50	F	1.63	55	20.70082	0.000914	0.0081261	0.0139521	N/A
38	40-50	F	1.57	54	21.90758	0.002851	0.0006296	0.019462	N/A
39	50-60	F	1.53	57	24.34961	0.002625	0.0068028	0.0312146	6.136
40	50-60	F	1.5	47	20.88889	0.002739	0.00408	0.0339054	N/A
41	40-50	F	1.6	65	25.39063	0.000292	0.0051109	0.0163632	6.2
42	50-60	F	1.67	51	18.28678	0.001512	0.0061444	0.0054143	4.400865
43	50-60	F	1.59	58	22.94213	0.000794	0.0160813	0.0275414	2.437536
44	40-50	F	1.6	73	28.51563	0.00155	0.018981	0.0566803	7.558398
45	30-40	F	1.56	54	22.18935	0.001332	0.0052964	0.1131605	3.234554

Adipose Tissue Microarrays Used in the Study

number	Age	Sex	Height (m)	Weight (kg)	BMI	*LINK-A* expression (scWAT)	*LINK-A* expression (VAT)
46	60-70	F	1.58	55	22	0.00001394	0.00005223
47	40-50	F	1.56	56.7	23.3	0.0001983	0.000004327
48	60-70	F	1.56	64	26.3	0.0004575	0.00004717
49	30-40	F	1.63	59	22.2	0.000001446	0.00001199
50	40-50	F	1.64	75	27.9	0.000019	0.00002397
51	40-50	F	1.61	53	20.4	0.000004771	4.74566E-05
52	80-90	F	1.45	70	33.3	0.0006986	0.000099413
53	50-60	F	1.55	49	20.4	0.0001618	0.00004101
54	30-40	F	1.67	62.5	22.4	0.00008105	0.000002567
55	40-50	F	1.58	66	26.4	0.0005373	0.0001225
56	20-30	F	1.63	62	23.3	0.0000301961	0.0000000968
57	30-40	F	1.63	60	22.6	0.0000195398	0.0000001573
58	30-40	F	1.60	58.5	22.9	0.0000435141	0.0000001278
59	50-60	F	1.57	52	21.1	0.0000168279	0.0000004449
60	50-60	F	1.60	65	25.4	0.0010318467	0.0000000226
61	40-50	F	1.53	54	23.1	0.0000074659	0.0000001112
62	30-40	F	1.64	58	21.6	0.0000051467	0.0000003146
63	50-60	F	1.58	54	21.6	0.0000251157	0.0000003146
64	60-70	F	1.57	49	19.9	0.0000144816	0.0000010221
65	40-50	F	1.59	65	25.7	0.0006975615	0.0000000183

Table S2. Reagent or Resource

Table S2. Reagent or Resource		
**Reagent or Resource**	**Source**	**Identifier**
**Antibodies**		
anti-vinculin	Cell Signaling Technology	Cat#13901
anti-HIF1α	Cell Signaling Technology	Cat#14179
anti-β-actin	Cell Signaling Technology	Cat#4970
anti-IL-1β	ABclonal	Cat#A11369
anti-CXCL16	HUABIO	Cat#ER1906-85
anti-UCP1	HUABIO	Cat#HA500188
anti-Phospho-PKA R2 (S99)	HUABIO	Cat#ET1610-29
anti-PKA	HUABIO	Cat#ET1704-15
anti-Phospho-HSL(S853)	HUABIO	Cat#ET1611-19
anti-HSL	Santa Cruz Biotechnology	Cat#sc-74489
**Reagents**		
Liposomal Transfection Reagent	YEASEN	Cat#40802ES01
DMEM	GibcoBRL	Cat#11965
FBS	GibcoBRL	Cat#16170
Pen/Strep	GibcoBRL	Cat#10378
insulin	Sigma-Aldrich	Cat#I5500
IBMX	Sigma-Aldrich	Cat#I5879
T3	Sigma-Aldrich	Cat#T2877
Rndomethacin	Sigma-Aldrich	Cat#I7378
Rosiglitazone	Sigma-Aldrich	Cat#R2408
NE	Supelco	Cat#N-069
IL-1β inhibitor	MCE	Cat#VX-765
CXCL16 inhibitor	Selleck	Cat#GI254032
Protease inhibitor	Roche	Cat#4693116001
TRIzol	Invitrogen	Cat#15596026CN
HiScript II qRT SuperMix	Vazyme	Cat#R222-01
SYBR qPCR Master Mix	Vazyme	Cat#Q711-012
Protein A/G PLUS-Agarose	Santa Cruz Biotechnology	Cat#sc-2003
**Critical Commercial Assays**		
Human IL-1β ELISA kit	absin	Cat#abs159986
Human CXCL16 ELISA kit	R&D Systems	Cat#DCX160
Mouse IL-1β ELISA kit	absin	Cat#abs520001
Mouse CXCL16 ELISA kit	R&D Systems	Cat#DY503
Dual-Luciferase Reporter Gene Assay Kit	YEASEN	Cat#11402ES60
BCA	Beyotime	Cat#P0009
SuperKine™ West Femto Maximum Sensitivity Substrate	ABBKINE	Cat#BMU102-CN
Histostain-Plus IHC Kit, Rabbit Primary	NeoBioscience	Cat#ENS004
**Biological Samples**		
Fresh frozen breast tissues	Huzhou First People's Hospital	2021kyII055
Fresh frozen adipose tissues	Affiliated Hangzhou First People's Hospital, Zhejiang University School of Medicine	ZN-20230918-0211-02

**Bacterial and Virus Strains**		
DH5a Competent Cells	Thermo Fisher Scientific	Cat#18265017
**Experimental Models: Organisms/Strains**		
Human *LINK-A* Knock in TG mice	Cyagen Biosciences Inc. (Guangzhou, China).	N/A
C57BL/6	Cyagen Biosciences Inc. (Guangzhou, China).	N/A
**Mouse feed**		
ND	Xietong Shengwu	Cat#1010063
HFD	Research Diets	Cat#D12492i
**Oligonucleotides**		
Oligonucleotides for qPCR, RNA interference	This paper	See Table S3
**Software and Algorithms**		
GraphPad Prism Software	GraphPad software	https://www.graphpad.com/
Leica Application Suite X	Leica Microsystems	https://www.leica-microsystems.com
ImageJ Fiji	National Institutes of Health	https://fiji.sc/
FastTree	Lawrence Berkeley National Lab.	http://meta.microbesonline.org/fasttree/

Table S3. Sequences of Oligonucleotides for qPCR and RNA Interference

**Table S3. Sequences of Oligonucleotides for qPCR and RNA Interference**		
**Oligonucleotides Resource**	**Sequence**	**Application**
LINK-A F1	AAGCACGTTTCCGACTTGAGTTG	Genotype PCR primer
LINK-A R1	GGGTGAGCATGTCTTTAATCTACC	Genotype PCR primer
LINK-A R2	CTCCTGACTACTCCCAGTCATAGC	Genotype PCR primer
Mouse Ucp1 qF	TTCAGGGAGAGAAACACCTGC	qRT-PCR primer
Mouse Ucp1 qR	CCTTCACGACCTCTGTAGGC	qRT-PCR primer
Mouse Cidea qF	ATCACAACTGGCCTGGTTACG	qRT-PCR primer
Mouse Cidea qR	TACTACCCGGTGTCCATTTCT	qRT-PCR primer
Mouse Ppargc1a qF	CCAGCCTCTTTGCCCAGAT	qRT-PCR primer
Mouse Ppargc1a qR	GTCGCTACACCACTTCAATCCA	qRT-PCR primer
Mouse Ppargc1b qF	AACCCAACCAGTCTCACAGG	qRT-PCR primer
Mouse Ppargc1b qR	ATGCTGTCCTTGTGGGTAGG	qRT-PCR primer
Mouse Cox8b qF	GAACCATGAAGCCAACGACT	qRT-PCR primer
Mouse Cox8b qR	GCGAAGTTCACAGTGGTTCC	qRT-PCR primer
Mouse Cxcl16 qF	TCCTTTTCTTGTTGGCGCTG	qRT-PCR primer
Mouse Cxcl16 qR	CAGCGACACTGCCCCTGGT	qRT-PCR primer
Mouse Il1β qF	AGCTTCCTTGTGCAAGTGTCT	qRT-PCR primer
Mouse Il1β qR	GCAGCCCTTCATCTTTTGGG	qRT-PCR primer
Mouse Il6 qF	TCCTCTCTGCAAGAGACTTCC	qRT-PCR primer
Mouse Il6 qR	GTCACCAGCATCAGTCCCAA	qRT-PCR primer
Mouse TnfαqF	GTTCTATGGCCCAGACCCTCAC	qRT-PCR primer
Mouse TnfαqR	GGCACCACTAGTTGGTTGTCTTTG	qRT-PCR primer
Mouse F4/80 qF	CTTTGGCTATGGGCTTCCAGTC	qRT-PCR primer
Mouse F4/80 qR	GCAAGGAGGACAGAGTTTATCGTG	qRT-PCR primer
Mouse Cd68 qF	CTTCTGCTGTGGAAATGCAA	qRT-PCR primer
Mouse Cd68 qR	AGAGGGGCTGGTAGGTTGAT	qRT-PCR primer

Mouse Nos2 qF	CCCTTCCGAAGTTTCAGCAGC	qRT-PCR primer
Mouse Nos2 qR	GGCTGTCAGAGCCTGGCTTTGG	qRT-PCR primer
Mouse Hbegf qF	CGGGGAGTGCAGATACCTG	qRT-PCR primer
Mouse Hbegf qR	TTCTCCACTGGTAGAGTCAGC	qRT-PCR primer
Mouse Ldha qF	TCTCGGATGTTGTGAAGGTG	qRT-PCR primer
Mouse Ldha qR	CTGCAGCTCCTTCTGGATTC	qRT-PCR primer
Mouse Pdk4 qF	TCTACAAACTCTGACAGGGCTTT	qRT-PCR primer
Mouse Pdk4 qR	CCGCTTAGTGAACACTCCTTC	qRT-PCR primer
Mouse Hk2 qF	CGTGTCCCTACCTTTGGGTT	qRT-PCR primer
Mouse Hk2 qR	CCAGGTCAAACTCCTCTCGC	qRT-PCR primer
Mouse Pdk1 qF	AGGATCAGAAACCGGCACAAT	qRT-PCR primer
Mouse Pdk1 qR	GTGCTGGTTGAGTAGCATTCTAA	qRT-PCR primer
Mouse Lox qF	ACTTCCAGTACGGTCTCCCG	qRT-PCR primer
Mouse Lox qR	GCAGCGCATCTCAGGTTGT	qRT-PCR primer
Mouse Hif1α qF	ACCTTCATCGGAAACTCCAAAG	qRT-PCR primer
Mouse Hif1α qR	ACTGTTAGGCTCAGGTGAACT	qRT-PCR primer
Mouse Vegfa qF	CAAAAACGAAAGCGCAAGAAA	qRT-PCR primer
Mouse Vegfa qR	CGCTCTGAACAAGGCTCACA	qRT-PCR primer
Mouse Glut1 qF	GCCAGCCAAGCTCATCAATG	qRT-PCR primer
Mouse Glut1 qR	GAGGCAGTCGGACATGCTC	qRT-PCR primer
Mouse 36B4 qF	AACGGCAGCATTTATAACCC	qRT-PCR primer
Mouse 36B4 qR	CGATCTGCAGACACACACTG	qRT-PCR primer
Mouse Gapdh qF	GTTGTCTCCTGCGACTTCA	qRT-PCR primer
Mouse Gapdh qR	GGTGGTCCAGGGTTTCTTA	qRT-PCR primer
Mouse 18S qF	ACAGGATTGACAGATTGA	qRT-PCR primer
Mouse 18S qR	TATCGGAATTAACCAGACA	qRT-PCR primer
Human UCP1 qF	GCTCCAGGTCCAAGGTGAATG	qRT-PCR primer
Human UCP1 qR	CAATGAATACTGCCACTCCTCCA	qRT-PCR primer
Human CIDEA qF	CATGTATGAGATGTACTCCGTGTC	qRT-PCR primer
Human CIDEA qR	GAGTAGGACAGGAACCGCAG	qRT-PCR primer
Human PPARGC1A qF	TGTGCAACTCTCTGGAACTG	qRT-PCR primer
Human PPARGC1A qR	TGAGGACTTGCTGAGTGGTG	qRT-PCR primer
Human PPARGC1B qF	GCTCTCCTCCTTCTTCCTC	qRT-PCR primer
Human PPARGC1B qR	ATAGAGCGTCTCCACCATCC	qRT-PCR primer
Human CXCL16 qF	GCCATCGGTTCAGTTCA	qRT-PCR primer
Human CXCL16 qR	CAATCCCCGAGTAAGCAT	qRT-PCR primer
Human IL-1B qF	TTCGACACATGGGATAACGAGG	qRT-PCR primer
Human IL-1B qR	TTTTTGCTGTGAGTCCCGGAG	qRT-PCR primer
Human HB-EGF qF	GGACCGGAAAGTCCGTGAC	qRT-PCR primer
Human HB-EGF qR	CCCGTGCTCCT CCTTGTTT	qRT-PCR primer
Human HIF1αqF	AAGTCTGCAACATGGAAGGTAT	qRT-PCR primer
Human HIF1αqR	TGAGGAATGGGTTCACAAATC	qRT-PCR primer
Human VEGFA qF	ACCATGAACTTTCTGCTGTCTTG	qRT-PCR primer
Human VEGFA qR	ATGGCTTGAAGATGTACTCGATCTC	qRT-PCR primer
Human GAPDH qF	GTCTCCTCTGACTTCAACAGCG	qRT-PCR primer
Human GAPDH qR	ACCACCCTGTTGCTGTAGCCAA	qRT-PCR primer
Human ACTIN qF	CACCATTGGCAATGAGCGGTTC	qRT-PCR primer
Human ACTIN qR	AGGTCTTTGCGGATGTCCACGT	qRT-PCR primer

Human 18S qF	GGACACGGACAGGATTGACA	qRT-PCR primer
Human 18S qR	GACATCTAAGGGCATCACAG	qRT-PCR primer
LINK-A qF	TCCTGCTTCTCTCACCCTTC	ChIP-qRT-PCR primer
LINK-A qR	CTGTTTGTAGAGCCTCCACA	ChIP-qRT-PCR primer
HB-EGF qF	GAGACCCCTGGTATGGGG	ChIP-qRT-PCR primer
HB-EGF qR	GAGAGGGCGGCGTTCACAAT	ChIP-qRT-PCR primer
IL-1β qF	TGGAGGCAAAACAGAGGAGTC	ChIP-qRT-PCR primer
IL-1β qR	TGGTGTCTGGTCCTGCAGAT	ChIP-qRT-PCR primer
CXCL16 qF	CCATGCTCATCCGTCACAAG	ChIP-qRT-PCR primer
CXCL16 qR	CCACATTTGTCTCTCGCTGC	ChIP-qRT-PCR primer
LINK-A LNA	TGGATAAATGAGCTGT	RNA interference
Scr LNA (Negative control)	AACACGTCTATACGC	RNA interference
shHIF1α#1 F	CCGG AAGCCGCTGGAGACACAATCA CTCGAG TGATTGTGTCTCCAGCGGCTT TTTTTG	RNA interference
shHIF1α#1 R	AATTCAAAAA AAGCCGCTGGAGACACAATCA CTCGAG TGATTGTGTCTCCAGCGGCTT	RNA interference
shHIF1α#2 F	CCGG A AGGAAGAACTATGAACATAA CTCGAG TTATGTTCATAGTTCTTCCTT TTTTTG	RNA interference
shHIF1α#2 R	AATTCAAAAA A AGGAAGAACTATGAACATAA CTCGAG TTATGTTCATAGTTCTTCCTT	RNA interference

Table S4. Sequences of Promoter

**Table S4. Promoter sequence**
***LINK-A* promotor sequence(2300bp):**
TGGAATTCAAGCTGTGGGTGAGAAGCTCTCTAGCAGGGACTCTGACCTTATGGAGGATCGCTGTTTCCCCCATTTTTCCTTTTCACC CAAAAAAGTCCTGCTTCTCTCACCCTTCAAACAGCCTGTGAGCCTAAATTTTTGTGGCCATGGGACAGACAAGGACCCCGTCTTCA GCTGAACTAAGGAAAAGTCCTGCGACATCTTTGGTACGCACTGTGGAGGCTCTACAAACAGCCATCAAACTCCAACCAGTCACCCA ACCAGAGCCTCTGAGGATGGCCCCTTCTGCGGGGAACCCTTGGAGCCAGAATCCCCAGCCCAGGACCGAGCCCTGCTCAGGGGCC TACAGGACAGCCCAGCAAGACACCTGCACATTACATCTCCTTATCTCTCTCTGGGTCCCTGGAGGGAACATCCCAGGAGGGCAGGA TCAGGGCTGTCCCTTTTGCCCAAAGCCTTCCACTGTGGCTGGCGCTCAGGAGGTGCGCATGGGGTTGTGCCAGCTTCCTGGAACTG CCATTACAGGTTGCCACACACCAAGGGGCTCCAAACAACATCCTCTCACAGTTCTGGAGGCCAGATCCAAAACCCAGGTGTCAGC AGGCCTGCTTCCTCCCGGAGGCCCTGAGGGAGAGCCCACTCCAGCAGCTCCGACGTCTGAGGGCTGCCAGCAGCCCTCGGGATAC CAACTTCGGCCTCATCCTCACAGTGGCTCTTCCTTTGTGTCTCCAGGTCTCCCTCTCCTTTCCCTTTTAAGGACACAGACACTGGACT TATGGCCCAACCTACTCCGGCATGACTCTAGCTTAACTTACTTACATCTGCCAAGACCCTATTCCCAAATAAGGTCACACTCACGGGT AACTTGGGCTAGGACTTCAACATACCCCCGTGGGGGCCACGATTCAACCCCCTACACGATCGACTGAAGGAAGGAACGTGGTCCG AGCCCCAGGAGGAATCTTCTCAGGTGCTCAGCCTCCCTGGACCTTGCCAAGGCCGCCCCCAGTGCCTCCTGCGCAGGAAGAATGA GCACATAGGTGGGGCTGAGGAGGCTGCAGGGGGCCTTCCCTGAGCATCCGTCCACTCCCCGTCTGTCCACTCCCTGCACCTGGACT CAAACAATGGAAGGAAGCACTTGGTCATATCCATGATCGCGTCCCAAACAGTGTGGGAATACATGCGAGGGGTTGGATGTGCTTGT GAGGAATGGGTACGTGGAGGTGTGTGCATGGGGTGTGATATGTGTGTGGAGGGGGGGTGTGTGTGGTGTGTGTGTGGTGTGATGTG TGTGTAGTGTGCAGTGTGTGTGCTGTGTGCTGTGTGGTGTGCAGTGTGTGTGCTGTGGTGTGTGTGTGGTGTGCAGTGTGTGGTTTG TGTGTGTGGTGTGTGCTGTGTGTGGTGTGCAGTCTGTGGTGTGTGTGTGGTGTGCAGTGTGTGTGTATACTGTGCAGTGTGTGGTGT GTGCTGTATGTGATGTGTGGTGTGCAGTGTGTGTGGTGTGTGTAGTGTCCAGTGTGTGTGGTGTGTGGTGTGCAGTGTGTGGTGTAT GGTGTGCAGTGCTGTATGTAGTGTGTGTGTAGTGTACAGTTTGGTGTGTGGTGTGTGCTTTGTGGTGTGCAGTGTGCATGTGTAGTG TGCAGTGTGTGGTGTGTTGTGTGCTGTATGTGGTGTGTGGTGTGCAGTGTGCATGTGTAGTGTACAGTGTGTGTGGTGTGTGGTGTG CAGTGCTGTATGTAGTGTGTGTGCAGTTTGGTGTGTGCTGTGTGGTGTGTGGTGTGCAGTGTGCGTGTATAGTGTGCAGTGCGCAGT GTGTGGTGTGCTGTGTGTGTGGTATGTGTAGTGTGCAGTGTGTTCTGTGTGTAGTGTATGGTGTGTGGTGTGCAGTGTGCATGTGTA GTGTGCAGTGCGTGTGGTGTGGTGTGCAGTATGTGTGGTGTGTGCTGTGTGTGGTATGCAGTGTCTGTGGTGTGTGGTGTGCAGTGT GTGTGCTGTGTGTGGTGTGCAGTGTGTGTGGTGTGTTTGTGTAGTGTAGTGTGTGTGGTGTGTGGTTTGCAGTGTGTGTGGTGTGCA GTGTGTGTGGTGTGTTCTGTGTGTGGTGTGTGGTGTGTGCTGTGTGTGGCATACAGTGTGTGTGGTGTGTTTGTGTAGTGTGCAGTG AGTGCGGTGTGTGCTGTGTGGCGTGCAGTGTGTGTGGTGTGTGGTGTGCAGTGTTGTATGTAATGTGTGTGTAGTGTGCAGTTTGGT GTGTGGTGTGTAGTGTGCAGTTTGGTGTGTGGTGTGTGCTGTGTGTGGTGTGT
***LINK-A* promotor truncated sequence:**
TCCTGCTTCTCTCACCCTTCAAACAGCCTGTGAGCCTAAATTTTTGTGGCCATGGGACAGACAAGGACCCCGTCTTCAGCTGAACTA AGGAAAAGTCCTGCGACATCTTTGGTACGCACTGTGGAGGCTCTACAAACAG
***LINK-A* promotor mutated truncated sequence:**
TCCTGCTTCTCTCACCCTTCATTATACCTGTGAGCCTAAATTTTTGTGGCCATGGGACAGACAAGGACCCCGTCTTCAGCTGAACTAA GGAAAAGTCCTGCGACATCTTTGGTACGCACTGTGGAGGCTCTACATTATA

HB-EGF promotor sequence(2300bp):

ATGAAGCTGCTGCCGTCGGTGGTGCTGAAGCTCTTTCTGGCTGCAGTTCTCTCGGCACTGGTGACTGGCGAGAGCCTGGAGCGGCT
TCGGAGAGGGCTAGCTGCTGGAACCAGCAACCCGGACCCTCCCACTGTATCCACGGACCAGCTGCTACCCCTAGGAGGCGGCCGG
GACCGGAAAGTCCGTGACTTGCAAGAGGCAGATCTGGACCTTTTGAGAGTCACTTTATCCTCCAAGCCACAAGCACTGGCCACACC
AAACAAGGAGGAGCACGGGAAAAGAAAGAAGAAAGGCAAGGGGGTGTCAGGAAGTGTTTCTCTCCACCCTCCCCTGGGGAGAG
CCTTGACCCCAAGGTGGCTTTGTTTTGGGGAAGCAGGTGGCCAGGCAGCTGCTGAGGGAGGGCAAAGTCATGGCTTGGGCGGTGA
CCATGCACTGAGGTCCTAGGCCCGATACTCATCACCCAAAAGGATAATCTAACAGTGGCTCTTAGCCAAATAGGTGACAGGTGAGCT
TCTGTCTGAAACCAGCCCAACTATATCATAAAGCCTCAGATATGGCCTAGGGGAAGGCAAGCGATCTAGAGAAGTGAGAGAAAAGA
GGCCTCTAAGGAAATAGAGTGGTCAAGTGCTTAGTGATTATTATTAACAGCAACAATAATAATGGGTCACATTTATTGAGAGCTTATA
GTGTGCCTGGCACTATACTAACTACTTTTATGTGTTCCCGCATTCATTTCTCAAAAAGCTCCCTACAAAGTAGTCATTATTATTATTGTT
ATTATTTTTCCATTTGAGAGACGTCAAAACTGAGGCTCAGTGAAATTAAGTTTCTTGTCCAAGGTCACACAGTAAGTAGCAAAGGC
AGGATTCAAACTCAGGTCTGTAGTGATTCCAGTGCACTATGGAAACCTAGGCCTAGGTTACTGGAGCAAGTTAAGAGGGCAGGGCC
AGACTGACCCCTGCAAAGGTGGGGATAGGGAGGGACAGTGGGATAGACTGTGTATAAATAGCAGGCCTGTTTTTATCCCTACACAC
AGGCAGAATCCGGCAAGTGCTCTATCTGGCACAATTTCAGGGCTCTGAAAGCCAACCCAGCCCTGTGTCAAACAACTCTCTCCCCA
GCACGTCCCTGGAAACTGAAATACCTACTGAGACACTCATTTCTTTGGGTCAGGACAGTGACTCCAGAAGACCCCTGCAAAAGCAC
ATGGGTTGGAAGAGAAACACCTAGTGTCTGTGAAACAGCAATCTAGGTTAGCCAGCTGGGTCCTATGGGAGGGTGGCTCATTTTTT
ATGTTGAGGAAAAAGGTGGCAGTCAAAGGCAAGGAAAAGAATAAGGCTTGTTTCACAACCTGTGTCTCTCCAATCTCAGAAGCCT
GGGAATTACAACCCATAGTTCAAAAATCCTAATGTCCCCTGAGGGAAGATGGGAGGAAGCCACTCCAGAGACTAAGAGAGTACTAA
CTTCCTTTGCTTTTGCTGGATTGATACTAAGTGCCTGCAACTTCAACTCTGGTTATCTCGGGTTTATGAGAACGGCTGGCATTCACGT
GTCTAATTTTTAAATGATAGATGAATTAATTATTATTTGATACTTGCCGTGTTGGTAGGCCAAAAGGTGGGCTTCTGAAACTTAGAGG
GTGACAGGGATGGAGACCCCTGGTATGGGGCTTAGATGAAGCAAGAGGCCAGGCCCGGAACAGAACTCAGCTCAGCAGTCAGTCA
CAAGGCTGCAGAGTGACAGGCGTTTGGGGAAAGGTAGGAACCGCTCTCCGCCACCTGCCGGTCAGTAGCCCTACCCACAATCAGG
CCGCGGCTGCCTCGGCCTGGTCCCAAAAATTGTGAACGCCGCCCTCTCCTCCCGCCCAGTCCTCCGCCGCGCCCGCGGGGTCGGGG
GCTGCGCTGCCGGCGCCGCGAGCCGGGCGAGCTTCCCAGCACGGGAGGAGGGAGGGGGCGGCGCCGGGCGGGGCGGAGCTTCC
GCGCCTCCTGGAGCCTTATTCCCGGCCGCGAGCCGGGCAGCGCTCATTCGGCCGAAGGAGCTACGCGGGCCACGCTGCTGGCTGG
CCTGACCTAGGCGCGCGGGGTCGGGCGGCCGCGCGGGCGGGCTGAGTGAGCAAGACAAGACACTCAAGAAGAGCGAGCTGCGCC
TGGGTCCCGGCCAGGCTTGCACGCAGAGGCGGGCGGCAGACGGTGCCCGGCGGAATCTCCTGAGCTCCGCCGCCCAGCTCTGGTG
CCAGCGCCCAGTGGCCGCCGCTTCGAAAGTGACTGGTGCCTCGCCGCCTCCTCTCGGTGCGGGACCA

HB-EGF promotor truncated sequence:

GAGACCCCTGGTATGGGGCTTAGATGAAGCAAGAGGCCAGGCCCGGAACAGAACTCAGCTCAGCAGTCAGTCACAAGGCTGCAG
AGTGACAGGCGTTTGGGGAAAGGTAGGAACCGCTCTCCGCCACCTGCCGGTCAGTAGCCCTACCCACAATCAGGCCGCGGCTGCC
TCGGCCTGGTCCCAAAAATTGTGAACGCCGCCCTCTC

HB-EGF promotor mutated truncated sequence:

GAGACCCCTGGTATGGGGCTTAGATGAAGCAAGAGGCCAGGCCCGGTTATAAACTCAGCTCAGCAGTCAGTTTAAAGGCTGCAGA
GTGACAGGCGTTTGGGGAAAGGTAGGAACCGCTCTCCGCCACCTGCCGGTCAGTAGCCCTACCTTAAATCAGGCCGCGGCTGCCTC
GGCCTGGTCCCAAAAATTGTGAACGCCGCCCTCTC

IL-1β promotor sequence(2300bp):

ATGGCAGAAGTACCTGAGCTCGCCAGTGAAATGATGGCTTATTACAGTGGCAATGAGGATGACTTGTTCTTTGAAGCTGATGGCCCT
AAACAGATGAAGTGCTCCTTCCAGGACCTGGACCTCTGCCCTCTGGATGGCGGCATCCAGCTACGAATCTCCGACCACCACTACAG
CAAGGGCTTCAGGCAGGCCGCGTCAGTTGTTGTGGCCATGGACAAGCTGAGGAAGATGCTGGTTCCCTGCCCACAGACCTTCCAG
GAGAATGACCTGAGCACCTTCTTTCCCTTCATCTTTGAAGAAGAAAGAAAGGAAGAAAGAAAGAAAGAAAGAAAGAAAGAAAGA
AAGAAAGAAAGAAAAGAAAAGAAAGAAAGAGAGAGAGAAAGAAAAAGAAAGAGGAAGGAAGGAAGGAAGGAAGAAAGACAG
GCTCTGAGGAAGGTGGCAGTTCCTACAACGGGAGAACCAGTGGTTAATTTGCAAAGTGGATCCTGTGGAGGCAAAACAGAGGAGT
CCCCTAGGCCACCCAGACAGGGCTTTTAGCTATCTGCAGGACCAGACACCAAATTTCAGGAGGGCTCAGTGTTAGGAATGGATTAT
GGCTTATCAAATTCACAGGAAACTAACATGTTGAACAGCTTTTAGATTTCCTGTGGAAAATATAACTTACTAAAGATGGAGTTCTTGT
GACTGACTCCTGATATCAAGATACTGGGAGCCAAATTAAAAATCAGAAGGCTGCTTGGAGAGCAAGTCCATGAAATGCTCTTTTTCC
CACAGTAGAACCTATTTCCCTCGTGTCTCAAATACTTGCACAGAGGCTCACTCCCTTGGATAATGCAGAGCGAGCACGATACCTGGC
ACATACTAATTTGAATAAAAATGCTGTCAAATTCCCATTCACCCATTCAAGCAGCAAACTCTACCACCTGAATGTACATGCCAGGCAC
TGTGCTAGACTTGGCTCAAAAAGATTTCAGTTTCCTGGAGGAACCAGGAGGAGCAAGGTTTCAACTCAGTGCTATAAGAAGTGTTA
CAGGCTGGACACGGTGGCTCACGCCTGTAATCCCAACACTTTGGGAGGCCGAGGCGGGCAGATCACAAGGTCAGGAGATCGAGAC
CATCCTGGCTAACATGGTGAAACCCTGTCTCTACTAAAAATACAAAAAATTAGCCGGGCGTGGCGGCAGGTGCCTGTAGTCCCAGC
TGCTGGGGAGGCTGAGGCAGGAGAATGGTGTGAACCCGGGAGGCGGAACTTGCAGGGGGCCGAGATCGTGCCACTGCACTCCAG
CCTGGGCGACAGAGTGAGACTCTGTCTCAAAAAAAAAAAAAAAAGTGTTATGATGCAGACCTGTCAAAGAGGCAAAGGAGGGTGT
TCCTACACTCCAGGCACTGTTCATAACCTGGACTCTCATTCATTCTACAAATGGAGGGCTCCCCTGGGCAGTACCCTGGAGCAGGCA
CTTTGCTGGTGTCTCGGTTAAAGAGAAACTGATAACTCTTGGTTGGTATTACCAAGAGATAGAGTCTCAGATGGATATTCTTACAGAA
ACAATATTCCACTTTTCAGAGTTCACCAAAAAATCATTTAGGCAGAGCTCATCTGGCATTGATCTGGTTCATCCATGAGATTGGCTA
GGGTAACAGCACCTGGTCTTGCAGGGTTGTGTGAGCTTATCTCCAGGGTTGCCCCAACTCCGTCAGGAGCCTGAACCCTGCATACC
GTATGTTCTCTGCCCCAGCCAAGAAAGGTCAATTTTCTCCTCAGAGGCTCCTGCAATTGACAGAGAGCTCCTGAGGCAGAGAACAG
CACCCAAGGTAGAGACCCACACCCTCAATACAGACAGGGAGGGCTATTGGCCCTTCATTGTACCCATTTATCCATCTGTAAGTGGGA
AGATTCCTAAACTTAAGTACAAAGAAGTGAATGAAGAAAAGTATGTGCATGTATAAATCTGTGTGTCTTCCACTTTGTCCCACATATA
CTAAATTTAAACATTCTTCTAACGTGGGAAAATCCAGTATTTTAATGTGGACATCAACTGCACAACGATTGTCAGGAAAACAATGCAT
ATTTGCATGGTGATACATTTGCAAAATGTGTCATAGTTTGCTACTCCTTGCCCTTCCATGAACCAGAGAATTATCTCAGTTTATTAGTC
CCCTCCCCTAAGAAGCTTCCACCAATACTCTTTTCCCCTTTCCTTTAACTTGATTGTGAAATCAGGTATTCAACAGAGAAATTTCTCA
GCCTCCTACTTCTGCTTTTGAAAGCCATAAAAACAGCGAGGGAGAAACTGGCAGAT

IL-1β promotor truncated sequence:

TGGAGGCAAAACAGAGGAGTCCCCTAGGCCACCCAGACAGGGCTTTTAGCTATCTGCAGGACCAGACACCA

IL-1β promotor mutated truncated sequence:

TGGAGGCAATTATAAGGAGTCCCCTAGGCCACCCAGACAGGGCTTTTAGCTATCTGCAGGACCAGACACCA

CXCL16 promotor sequence(2300bp):

ATGGGACGGGACTTGCGGCCCGGGTCCCGCGTGCTCCTGCTCCTGCTTCTGCTCCTGCTGGTGTACCTGACTCAGCCAGGCAATGG
CAACGAGGGCAGCGTCACTGGAAGTTGTTATTGTGGTAAAAGAATTTCTTCCGACTCCCCGCCATCGGTTCAGTTCATGAATCGTCT
CCGGAAACACCTGAGAGCTTACCATCGGTGTCTATACTACACGAGGTTCCAGCTCCTTTCCTGGAGCGTGTGTGGAGGCAACAAGG
ACCCATGGGTTCAGGAATTGATGAGCTGTCTTGATCTCAAAAGCTCTCCAGCTCTCTAAATGCAGTTGGGGGGGAGATGGCATCAG
GCTGGAGGAGGTGCTTGGGAATCCAGGGCCCGAGTCCTCCCCAGCCTCAGATACCAGATTTCTATACCGATGCAGCCGGGCATCTCC
CACAGTAAGCTGTCGGGGCTTTCGGGGATCCCCAGAGCCCATGGGACAGGCCATGCTGTGACAGAGGAGAGCATAGGCCCAAGGA
GCCAGGTCCTTGGTCCCTGAGCCCCAGCTTGCGGTTCCCTGGGCTCCATCCACTAGCAGATCAATGCCCAAGATGGTGCCATGCTCA
TCCGTCACAAGTAAGAGACAGGAAACGTGCAGGGGGGCAGCCTCTGGGGAAAGAGGGAAAAGTTGAGGTGGTAGGGGGCAGCG
AGAGACAAATGTGGTCAGGGTCAGGAGTACCAAACTCAAATACCTACGGGGCTAGGCAAGTAACCTCACTGAGTAAAGGGCTCTG
GGTAGAGAGGGCACGCTCCTTCTAAAGGGGACAGCTGCTACAGAGGACCAGAATGCGCTGCCATGCAGAAATGTAGGCCCACTGT
CAGAGCTTGAGATTTTTCTTGGGAATGTGTAAATCCAGACTTCTGGATACTTTTTTTCTTGCAATGTTTAAACATTAAATTAAAAAAA
AAAACCCCACTGTGGGTCAATAAAAACACCTTTTCAGGCCAGATTGGCCTGAAGGCCAGCACTCAGAACTAGTGGCAGGGGAGGG
CTTCCGAAGGGAGGATGGCTGGGCAAGGGGTAGGGCAGGGCCAGCCCTGGGTTCTTACCACTCCTCTGTCCCTTCCTCTTCTGGGG
TCTTGTTTCGTTCTCCACCCTGTCCTCTCGGCAGCCATCCTTGCCACCTCCTGCCTCCCGGGCAGCCTCCTCTGACTCTCCTGGAGG
GTTTGTTTCCTGGGGGGACTCCCTGCTGGTAGGGGCTAGCTCCCGGTCCTCCTCTGGTTCCACCTTCTCTGTGCTGCCTGCACCCCC
GTCCTCTCTCTTCTCTTCTTCTTCATCTTCCTCCTCTTCCTCCTCCCCTTCCTCCCCATCCTCTAGGCTGACAAAGCTGATGTAGGGGC
TCAGGTCTCGCAGGTGGAGTGGAGGGTTGTCTCCCAGGAGCCAGGCAGTCCCCAGGCCTGGTCCAGGACCTGGTTCTGCCCCTTG
CCCCAGGCTGATGCCCACACTATGGTTGGGCAGGACATGGAGCACCCAAAGGGCAGGGTCCTGGGGTAGCCTTCTGATCTGGGCCT
CCAGCTGCCGGCAGCGGCCCTCAAGGCTAGTCCCTACAGCTCCACGCTCTGCCACAAACTTTCGGAACCAGCCGAAGAGAAGGGC
AGTGAAATCAAGGAACTCATCCCGGTATCCAGACACAAACTCCATGTCTTCAGCGGCACAGGGCCCCGGCCCAGACTGAGCAAAA
GAAGGGATATATGGTGAGGGACAGGAGAGCAATGATGGAGTTTGGGGCTGGGTCACAAATTGAGGGAGAGGGTAGGTTTGAGAGT
CACTTAGGGTTGGATCCGTGGATATGCTAGGAGCGGAGAAGAGGATTTTTCAGGGCTGAGGATGGGAGTTTAGGGAGGAAAATGGG
GGCTGGACTTGAAAGTGGGTGACCAGGGTTGGAGAAGACTACTCAGGGATGAGATGGTATTTGGGGCTTAAAATTGGAGAGTTCA
CACCCTCAAAAGCAGAGTTTAAATTAAGGCTAGAGAGGAGAGAGGGCTAATCCTATCGGGAACGGTTTGGTGGGGAGGGAAGCAT
TTTGGATGCGTCTGTGGGGTATGGGAGTGGGAGCGAGGGGGTTCCCTGGGCTCGGATCTGCGTGGCGGGCAGCCCCGGTAGACCTA
GCCCCTCCCGGCCGCCCCACTCCTCCGGTACCTGCAGCCGCCACCACTTTTCGAAGGTAAATACCTCGCGGTGGAGGGTGCGCGGG
TGCGGTTGTGACGTCAGGGCTGCCCGAGCCCTCGCCGGCTCAGCGGGCGGCCACGCCCCCCGGGCTC

CXCL16 promotor truncated sequence:

CCATGCTCATCCGTCACAAGTAAGAGACAGGAAACGTGCAGGGGGGCAGCCTCTGGGGAAAGAGGGAAAAGTTGAGGTGGTAGG
GGGCAGCGAGAGACAAATGTGG

CXCL16 promotor mutated truncated sequence:

CCATGCTCATCCGTTTAAAGTAAGAGACAGGAATCTCTCAGGGGGGCAGCCTCTGGGGAAAGAGGGAAAAGTTGAGGTGGTAGGG
GGCAGCGAGAGACAAATGTGG
